# Performance changes and neuromuscular alterations in sprague dawley rats following repeated occupational exposures to the pesticide malathion

**DOI:** 10.1371/journal.pone.0357900

**Published:** 2026-09-15

**Authors:** Kaitlyn M. Miller, Dakota R. McMeans, Martha J. Sonner, David R. Ladle, Shannon H. Romer

**Affiliations:** 1 Naval Medical Research Unit Dayton, Environmental Health Effects Laboratory, Wright-Patterson Air Force Base, Dayton, Ohio, United States of America; 2 Wright State University, Dayton, Ohio, United States of America; 3 Leidos, Reston, Virginia, United States of America; 4 Oak Ridge Institute for Science and Education, Oak Ridge, Tennessee, United States of America; Fujita Health University, JAPAN

## Abstract

Organophosphate pesticides, such as malathion, have been linked to various pathological effects, including neurodegeneration. Acute exposure to malathion, a non-reversible acetylcholinesterase (AChE) inhibitor, causes adverse effects in mammals. However, the impact of subacute, occupational-like repeated exposure on motoneuron (MN) and neuromuscular junction (NMJ) anatomy is less understood. We hypothesized that repeated malathion exposure would produce motor performance deficits accompanied by anatomical alterations in spinal MNs and NMJs consistent with neurodegeneration. We also hypothesized that galantamine, a reversible AChE inhibitor, would provide neuroprotection. Adult male and female Sprague–Dawley rats received subcutaneous saline, malathion (50 mg/kg), galantamine (5.0 mg/kg), or both once daily, five days per week for four weeks. Animals were evaluated immediately after the final exposure and following a four-week recovery period. After exposure, no evidence of spinal astrogliosis and plasma cholinesterase activity was detected. Despite this, cholinergic (C-bouton) synaptic remodeling was observed on lumbar spinal MN somas in females and both sexes showed decreased open-field motor activity. Exposed males performed worse on the accelerod, while exposed females exhibited reduced grip strength. These functional deficits corresponded with a 25% decrease in hindlimb NMJ innervation in both sexes. After recovery, exposed rats showed increased NMJ fragmentation, suggesting remodeling during recovery. Galantamine treatment protected against health effects associated with repeated occupational-like malathion exposure, indicating involvement of cholinergic mechanisms despite minimal changes in plasma cholinesterase activity. Overall, repeated low-concentration malathion exposure is associated with locomotor deficits and peripheral neuropathy.

## Introduction

Pesticide use is necessary to protect crops and control the spread of vector-borne disease. However, the use of pesticides may pose a risk to human health. Pesticides and their metabolites can enter humans and animals directly via inhalation, ingestion, and dermal absorption, or indirectly as pesticides settle in soil and groundwater and enter the food chain. Although acute pesticide poisonings do occur, subacute repeated environmental and occupational exposures to pesticides are more common. These chronic or subchronic exposures to pesticides are associated with the slow development of health effects, cancers, neurological disorders, and other chronic diseases [1–4]. Most available research is focused on the effects of acute poisoning; thus, it is important to investigate the potential health effects associated with these more common occupational and environmental chronic and subchronic exposures.

Malathion (MAL) is one of the most widely used organophosphate (OP) pesticides in the United States [5,6] and is used residentially, agriculturally, and for public mosquito-borne disease control. Additionally, MAL is approved for use by the United States Military. The Navy and Marine Corps use MAL to target *Aedes* habitats necessary to protect Service Members from vector-borne diseases such as Dengue, Yellow Fever, and Zika [7]. Because of the widespread use, Military personnel are likely to be exposed to MAL. The use of MAL may also confer a risk to U.S. civilians. The United States Geological Survey’s Pesticide National Synthesis Project reports almost 1 million pounds (epest-high estimate) of agricultural MAL are used per year in the U.S. [8]. Thus, the knowledge of potential health risks associated with occupational and/or operational MAL exposure is critical so that these pesticides can be used to protect, and not harm, Service Members and civilians alike.

Once in the body of humans and animals, MAL is rapidly metabolized through oxidation by cytochrome P450 to malaoxon, a potent irreversible inhibitor of acetylcholinesterase (AChE) and the primary driver of toxicity [9–11]. The inhibition of AChE leads to the accumulation of the neurotransmitter acetylcholine at central and peripheral synapses, such as the neuromuscular junction, leading to prolonged cholinergic receptor activation. Cholinergic overstimulation can lead to seizures, muscle fasciculations, excessive salivation and lacrimation, miosis, and paralysis. The LD_50_ for oral ingestion in rats is > 5400 mg of MAL per kg of body weight, and >4000 mg of MAL per kg of body weight for dermal exposure. Also in rats, the No Observed Effect Level (NOEL) is equivalent to 350 mg of MAL per kg of body weight per day [12,13]. For humans, the lethal oral dose has been reported historically in a wide range of 350–2,000 mg of MAL per kg of body weight [14,15] and models have predicted the median LD_50_ around 3,655 mg of MAL per kg of body weight [16]. These relatively high values place MAL in a low toxicity classification [17]. However, cognitive and locomotor deficits have been reported in humans and animals from low-concentration MAL exposures without causing overt cholinergic symptoms or significant impacts on AChE activity [18–20]. This suggests that either alternative mechanisms or even minor impacts on AChE activity contribute to pathological effects in repeated exposure to low concentrations of MAL [21,22].

Despite known locomotor deficits, such as reduced motor coordination and muscle strength [23,24], no studies have examined the effect of repeated occupational MAL exposure on the structure of spinal motoneurons (MNs) and their projections to skeletal muscle at the neuromuscular junction (NMJ). The objective of this study is to investigate the impact of repeated occupational-like MAL exposure on locomotor behavior and motor unit structure. In addition, the role of cholinesterase inhibition was examined using the reversible cholinesterase inhibitor galantamine (GAL), an FDA-approved treatment for Alzheimer’s disease. Moreover, GAL has been proposed to be an effective countermeasure against OP poisoning [25], but has never been used to test for potential protective effects against subacute occupational exposures. To accomplish the objectives, adult male and female Sprague Dawley rats were exposed via subcutaneous injection to either 50 mg MAL per kg of body weight or saline once a day, five days a week, for four weeks (20 total exposures). Outcomes were assessed the week following the final MAL exposure and after a one-month recovery period. Neurobehavior was assessed with accelerod, open field motor activity, and a functional observation battery that includes grip strength assessment. Structural changes in spinal MNs and NMJs were assessed using immunohistochemistry and confocal microscopy. Despite the absence of a significant impact on plasma cholinesterase activity, we report functional locomotor changes as well as structural changes in the motor unit following repeated occupational-like exposure to MAL consistent with peripheral neuropathy. Moreover, we found sex-specific differences in both functional and structural impacts. Collectively, these findings demonstrate that performance-limiting health effects can occur at MAL exposure levels well below those associated with the canonical mechanism of OP-induced acetylcholinesterase inhibition.

## Materials and methods

This study was conducted under an approved animal use protocol in an AAALAC International-accredited facility in compliance with all federal statutes and regulations relating to animals and experiments involving animals and adheres to principles stated in the Guide for the Care and Use of Laboratory Animals, 8^th^ Edition, National Research Council (2011). A total of 48 adult male and 48 adult female Sprague Dawley rats from Charles River Laboratories (Wilmington, MA, USA) were used in this study. The total duration of the experiment was either 5 weeks for half rats or 9 weeks for the remaining rats (please see exposures and assessment timepoints). Rats were maintained with a 12-hour light/dark cycle and provided dry chow and water *ad libitum* in a temperature (20–26°C) and humidity (30–70%) controlled vivarium. Animals were monitored at least twice daily by trained personnel for signs of pain, distress, or illness. Humane endpoints were predefined and include >20% weight loss, severe clinical signs such as labored breathing, and any condition deemed moribund by the Attending Veterinarian. No animals exhibited signs of pain or distress, died unexpectedly, or reached predefined humane endpoints requiring euthanasia prior to the scheduled study endpoint. At study completion (either one week or four weeks following final exposure), animals were humanely euthanized in accordance with the American Veterinarian Medical Association Guidelines for the Euthanasia of Animals. Euthanasia was performed with exsanguination during transcardial perfusion under deep anesthesia (150 mg of sodium pentobarbital per kg of bw) administered via intraperitoneal injection. All efforts were made to minimize pain and distress.

### Exposures and assessment timepoints

Animals were randomly and evenly divided into four experimental groups: Saline control (SAL), MAL, GAL control, and MAL + GAL. The major route of MAL occupational exposure is dermal via handling MAL-containing powders and sprays or contact with contaminated clothing. Both MAL exposures and GAL treatments were administered via subcutaneous injection once a day, five days per week, for 4 weeks total to mimic dermal exposure in a controlled and consistent manner. Direct dermal application in rodents can present complications and confounds of cross-exposure and subsequent metabolism via other routes. Direct dermal exposure also has the potential for skin irritation. MAL injected subcutaneously is absorbed systemically and metabolized via the same pathways as dermal exposure. Moreover, there is precedent for this route of OP exposure in rodents in the literature, which will enable meaningful comparison of our results with other published findings (20, 105). MAL (Cat# 101182–088, VWR, Radnor, PA, USA) was diluted in 0.9% normal saline and administered at 50 mg of MAL per kg of body weight (bw). The MAL exposure concentration selected was consistent with other rodent studies that did not cause acute cholinergic symptoms but generated health effects similar to what has been observed in humans [20,24,26–31]. GAL (Cat# G02931G, Fisher Scientific, Waltham, MA, USA) was diluted in 0.9% normal saline and administered to the rats at a dose of 5.0 mg of GAL per kg of bw. This dose has been previously demonstrated to be therapeutically sufficient without causing adverse health effects in rats [32–35]. GAL was administered approximately 30 minutes prior to each MAL exposure. Approximately 30 minutes are required for GAL levels to elevate in the rat brain following subcutaneous injection [36]. Plasma levels of GAL are eliminated within 12–24 hours following a single bolus, with elimination faster in males than females. [34].

Two time points following the 4-week MAL exposure, termed immediate and delayed, were chosen for analysis. Animal groups analyzed within 1 week after the final exposure were termed immediate and abbreviated as iSAL, iMAL, or iGAL. The delayed time point was analyzed after a 4-week recovery following the last exposure (abbreviated as dSAL, dMAL, or dGAL). For neurobehavior, all 12 animals per experimental group were used for the immediate time point and 6 for the delayed time point. Following neurobehavioral tests, the rats were euthanized via exsanguination during transcardial perfusions under deep anesthesia (150 mg of sodium pentobarbital per kg of bw). Tissue collected from the rats was used for anatomical analysis with a 6 animals per sex per experimental group for the immediate time point and 6 animals per sex per experimental group for the delayed time point, totaling to 48 males and 48 females.

### Cholinesterase activity assay

Cholinesterase (ChE) activity levels were analyzed at both the immediate and delayed time points following the repeated exposure to MAL. Blood was collected during terminal procedures and processed to collect plasma and stored in a −80°C freezer. A commercially available AChE activity colorimetric assay Kit (Cat# ab287842, Abcam, Cambridge, UK) was then used to quantify plasma activity levels following the manufacturer’s instructions. Briefly, AChE in the sample reacts with the acetylcholine substrate to produce choline which is oxidized by choline oxidase to form the intermediate. The intermediate is probed to generate color, and the absorbance (OD 570 nm) is measured on a standard plate reader for quantification. Absorbance is measured three times at 10-minute intervals, then activity is calculated from changes in concentration over time. The units are expressed as a rate of choline production within a volume (nmol/min/mL), which indirectly, but proportionally, measures AChE activity.

### Behavioral assessments

Functional Observation Battery (FOB): FOB was used to evaluate and document the absence or presence of a set of behavioral and clinical signs. These observations were made while the rat was in the home cage, removed from the home cage, when handled, while the animal moved freely in an open field, and during manipulative tests. Cage side observations included examinations of posture, tremor, spasm, seizures, and/or convulsion. Observations while the rat is being held included assessing general reactivity, piloerection, muscle tone, lacrimation, salivation, fur appearance, porphyrin/facial crust, skin, and breathing. Manipulative observations include assessment of approach response, acoustic response, tail pinch response, visual placing, and righting reflex. One manipulative test of FOB is grip strength. During forelimb and hindlimb grip strength recordings, the animals’ paws were placed on a wire mesh (Chatillon Digital Forces Gauges DFIS10, Siemens, Chatillion, France) that the animals naturally held onto while gently being pulled backward. Forelimb grip strength was measured with the T-shaped adaptor, positioned parallel to the floor. Hindlimb grip strength was measured with the wire mesh screen adaptor angled slightly down (approximately 45°) toward the floor. Before the grip release, the maximum strength of the grip was recorded. There were three trials per animal, and the averages of the trials were recorded.

Accelerod: The test used a rotarod (AccuRotor EzRod, Omnitech Electronics, Inc., Columbus, OH, USA) with a modified cover to allow ample space for larger rats (>300g) to walk on the rotating rod. The animals were trained over two days to ensure introduction and ability to perform a minimum standard on a slow-moving rod at a constant speed of 11 rpm for 5 minutes. The animals were not exposed to acceleration on training days to keep them naïve to the test. For the test, the animals were removed from the cage and placed on an individual rod that was initially rotating at 4 rpm. The apparatus was set to accelerate from 4 to 40 rpm over 300 seconds. The test began when the acceleration started and ended when the animal fell off the rod or when the 300-second test concluded. The latency to fall, distance traveled, and final speed (in rpm) were recorded.

Motor Activity: Motor activity was analyzed using clear 16’ × 16’ polycarbonate open fields with an automated Photobeam Activity System (PAS, San Diego Instruments, San Diego, CA, USA). Two sets of stacked photobeams were used to track horizontal and vertical movements. The proprietary software (PAS, San Diego Instruments, San Diego, CA, USA) tracked and quantified fine movements (one photobeam break), locomotor movements (two photobeams broken in sequence), horizontal and vertical movements, distance traveled, active time, inactive time, total rears, percentage of time in the center, and speed. Recordings occurred for 30 minutes under controlled environmental conditions including noise, temperature, humidity, lighting, odors, and environmental distractions.

### Spinal cord immunohistochemistry, microscopy, and analysis

To prepare tissue for immunohistochemistry and microscopy analysis, rats were transcardially perfused with 4% paraformaldehyde in 0.1M phosphate buffer. The spinal cords were then removed and placed into 4% paraformaldehyde for two hours, PBS rinsed overnight and then stored in 15% sucrose at 4°C to cryopreserve. The spinal cords were transversely sectioned into 60-µm thick slices, using a freezing sliding microtome (Leica Biosystems SM2010 R, Clifton, NJ, USA) at the lumbar spinal level IV to V. Spinal cord sections were suspended in all wash and incubation solutions. Tissue slices were blocked in a 1:10 Normal Horse Serum (Cat# S-2000, Lot# ZH1108, Vector Laboratories, Newark, CA, USA) diluted in PBS with 0.1% Triton X-100. The primary and secondary antibodies used are listed in S19 Table in S1 File. The tissue was mounted on charged microslides in Vectashield mounting media (Cat# H 1000, Vector Laboratories, Newark, CA, USA). Using the Leica SP8 confocal microscope (Leica Microsystems, Deerfield, IL, USA), the tissue was imaged with a 20X objective lens (NA 0.75) at 10 × 1.0 μm z-steps with solid-state lasers. Grids of images were set up to capture the entire lamina IX of the ventral horn, and scans containing multiple images were stitched together for analysis. Consistent with other studies, MNs selected for quantification were differentiated from other ventral horn neurons by morphology and laminar location. Morphological characteristics used to discern α-MNs from surrounding cells included NeuN-IR, relatively large size, lamina IX localization, and large cholinergic inputs/C-boutons which were revealed by VAChT-IR [37–39].

Confocal microscopic image analysis included quantification of the MN C-bouton synapses, MN area, and spinal gliosis. With the MNs in lamina IX (S4 Fig), the C-bouton areas and densities on the MN were quantified by counting the number of C-boutons and measuring the mean cross-sectional areas for *en face* C-boutons using the Leica Application Suite X software (Leica Microsystems, Wetzlar, Germany). MN soma areas were measured in NeuN-positive cells using a single optical section containing the nucleolus. Assuming all somas fit a circular shape, MN diameters were mathematically derived from measured areas. MN diameters less than 20 µm were excluded from analysis, based on convention and historical reports of typical rat MN size [40]. Gaussian mixture modeling was used to assess modality of MN diameter distributions. The best-fitting model, selected by the lowest Bayesian Information Criterion, confirmed all groups were bimodally distributed. A cutoff value between small and large populations was defined by the intersection point separating the lower and upper fitted gaussian curve. Small, large, and mean MN diameters were then compared across all groups. Additionally, MNs exhibiting SK3-IR were analyzed as a distinct subpopulation to assess potential changes of SK3-positive cells. SK3 expression status was not factored into the previously described general MN size analysis. Lastly, spinal gliosis was analyzed by recording the integrated optical density and the mean intensity of the astrocytes (GFAP-IR positive) in lamina IX of lumbar spinal cord levels IV to V. The integrated optical density analysis was computed as pixel area multiplied by the mean intensity, using Image-Pro 10 (Media Cybernetics, Rockville, MD, USA) software.

### Whole muscle immunohistochemistry, microscopy, and analysis

Following euthanasia, the extensor digitorum longus (EDL) and soleus (SOL) muscles were dissected from the animals. The muscles were flattened between two glass slides in 4% paraformaldehyde solution for 2 hours, then washed and transferred to 0.01M PBS for storage. Muscles were incubated in normal horse serum (1:10 concentration) diluted in 0.01M PBS containing 0.3% Triton X-100 (PBS-TX 0.3%) for 90 minutes. Muscles were then incubated in PBS-TX 0.3% containing the primary antibodies overnight. The next day, muscles were washed with PBS-TX 0.3%, then incubated in PBS-TX 0.3% containing the secondary antibodies for 2–4 hours. The primary and secondary antibodies are listed in S19 Table in S1 File. Whole muscles are then mounted into a cover glass chamber, covered in ultrapure glycerol (Invitrogen, Waltham, MA, USA) and imaged using a Lecia SP8 confocal microscope (Lecia Microsystems, Deerfield, IL, USA) with a 20X objective lens (NA 0.75) at 1.0 µm steps. Large regions of the NMJ band, containing at least 20 NMJs, were captured for analysis in Image Pro 10 Software (Media Cybernetics, Rockville, MD, USA).

Denervation Analysis: Qualitative analysis of innervation was done using superimposed confocal images of NMJ bands along the EDL and SOL muscles with the Leica Application Suite X (Leica Microsystems). Each NMJ was categorized as one of three options (category I, II, or III). An NMJ was category (cat) I when the presynaptic NF-H fluorescent signal overlapped with most or all the postsynaptic nicotinic acetylcholine receptor (nAChR) signal. The NMJ was considered cat III if the NF-H signal was nearly or completely absented from the nAChR signal. If the NMJ was somewhere in between these two descriptions, meaning the NF-H covered around half of the nAChR signal, then the NMJ was cat II. The frequency of each innervation category was calculated for each individual muscle and averaged across each respective group.

Nicotinic Acetylcholine Receptor Anatomical Analysis: The post-synaptic side of the NMJ was represented by alpha-bungarotoxin fluorescent signal to label nAChRs. Using superimposed and flattened images of NMJ bands from EDL and SOL muscle, the nAChR-IR signal was isolated. In ImagePro (Media Cybernetics, Rockville, MD, USA) software, regions of interest (ROI) were drawn around 20 NMJs per muscle. A pixel intensity threshold filter was then used to isolate the nAChR-IR signal from the background signal within the area of a single NMJs. Data from both the entire ROI and the isolated nAChR-IR areas were used. Data collected from the entire ROI is considered a cross-sectional NMJ area.

Fragmentation Analysis: Using the same process described above to isolate the nAChR signal from the entire NMJ area, fragmentation could be assessed in 20 NMJs per muscle. Any NMJ containing more than one continuous nAChR-IR was considered fragmented. The thresholding filter described above was able to determine which areas of specified pixel intensity were distinguishable from another area. This outputs a number of subregions of nAChR signal within each ROI. A frequency of fragmentation was then determined in each muscle and averaged in each respective group.

### Figure composition

Microscope images were prepared by adjusting contrast and brightness in Leica Application Suite X software and always preserved all the information of the images. Graphs were composed in Origin Pro (OriginLab, Northampton, Massachusetts). All quantifications were carried out in original unprocessed images.

### Statistical analysis

All procedures and analyses were conducted by investigators blinded to experimental group. Data were analyzed in SigmaPlot (v14.5) and R. Distributional assumptions were evaluated using Shapiro–Wilk tests for normality and Levene’s tests for homogeneity of variance. Two-group comparisons were performed using Student’s t tests or Welch’s t tests and when variances were unequal nonparametric comparisons used Mann–Whitney tests were used. For comparisons involving more than two groups, one-way ANOVA with appropriate post hoc correction (Holm–Šidák, Tukey, or Dunnett) was applied when assumptions were satisfied, whereas nonparametric data were analyzed using Kruskal–Wallis tests with Dunn’s post hoc comparisons. Neurobehavioral outcomes involving multiple time points were evaluated using two-way ANOVA.

Hierarchically structured datasets were analyzed using mixed-effects or marginal modeling approaches to account for repeated measurements within animals. Because MN soma size spans a continuum of phenotypes rather than representing a homogeneous population, multiple MNs were sampled from each animal to characterize the distribution of MN sizes within each animal. Consequently, the individual animal, rather than the individual MN, served as the independent experimental unit for statistical inference, while within-animal correlation among MNs was accounted for analytically. MN diameter distributions were analyzed using linear mixed-effects models (REML estimation) with experimental group as a fixed effect and animal ID as a random intercept and generalized estimating equation (GEE) models with robust sandwich standard errors were used as complementary population-averaged estimates when mixed-effects models exhibited convergence limitations. Analyses were conducted on pooled MN populations and separately within small and large MN subgroups.

C-bouton area and density were analyzed using linear mixed-effects models to account for the nested structure of boutons within MNs and MNs within animals. Bouton area values were log-transformed to correct for right-skewed distributions prior to inference. NMJ area measurements were analyzed using Gaussian GEE models with animal ID treated as the repeated unit and an exchangeable working correlation structure. Because NMJ area distributions were right-skewed, analyses were performed on log-transformed values.

The relationship between nAChR fragmentation and NMJ area was assessed using a binomial logistic mixed-effects model with a logit link, including sex, muscle type, treatment group, and standardized NMJ area as fixed effects with full interactions and animal ID as a random intercept. NMJ area was standardized across the dataset (mean = 0, SD = 1) to facilitate comparisons of fragmentation probability across groups at equivalent relative NMJ sizes. Model adequacy was evaluated using convergence diagnostics and simulation-based residual analysis (DHARMa). Fixed-effect estimates are reported with Wald 95% confidence intervals, and pairwise contrasts were evaluated using Wald z tests.

Data from saline and MAL groups were included in GAL analyses to enable unified statistical comparisons. Statistical significance was defined as p < 0.05. Data are presented as mean ± SEM in figures and mean ± SD in text and tables unless otherwise indicated.

## Results

Throughout the study, the rats were monitored for signs of discomfort or cholinergic distress with daily observations and weekly body weight measurements (S1-S2 Tables in S1 File). No signs of distress were reported across any experimental group. Furthermore, there were no significant changes in body weight across experimental groups over the duration of the exposure and recovery periods (S3 Table in S1 File). Detailed clinical observations were performed with the Functional Observation Battery (FOB) at both the immediate and delayed time points (Dataset 1). No overt cholinergic symptoms were observed across all experimental groups (S1 Fig and S4-S5 Tables in S1 File); however, it was noted that some MAL-exposed rats had reduced muscle tone and felt flaccid when held following the final few exposures. Overall, the well-being of the animals was maintained throughout the duration of the exposure period in all experimental groups.

### Plasma cholinesterase activity is not reduced following repeated 50 mg/kg malathion exposures

OP poisoning is confirmed clinically with reduced blood cholinesterase activity levels. To examine the impact of repeated occupational MAL exposure on cholinesterase activity, blood plasma was collected at the immediate and delayed time points (S4 Table in S1 File). Although commercially available kits were used to monitor plasma AChE activity, the kits may also be detecting activity from other plasma cholinesterases, such as butyrylcholinesterase. At both the immediate and delayed time points, the males had no significant differences between the MAL and saline groups (S1 Fig and S5 Table in S1 File). Likewise, the females did not exhibit significant differences at the immediate and delayed time points between the MAL and saline experimental groups (S1 Fig and S5 Table in S1 File). Overall, daily 50 mg of MAL per kg of body weight did not significantly impact plasma cholinesterase activity at the immediate and delayed time points.

### Neurobehavior effects in accelerod, grip strength, and motor activity following repeated malathion exposures

**Fig 1:**
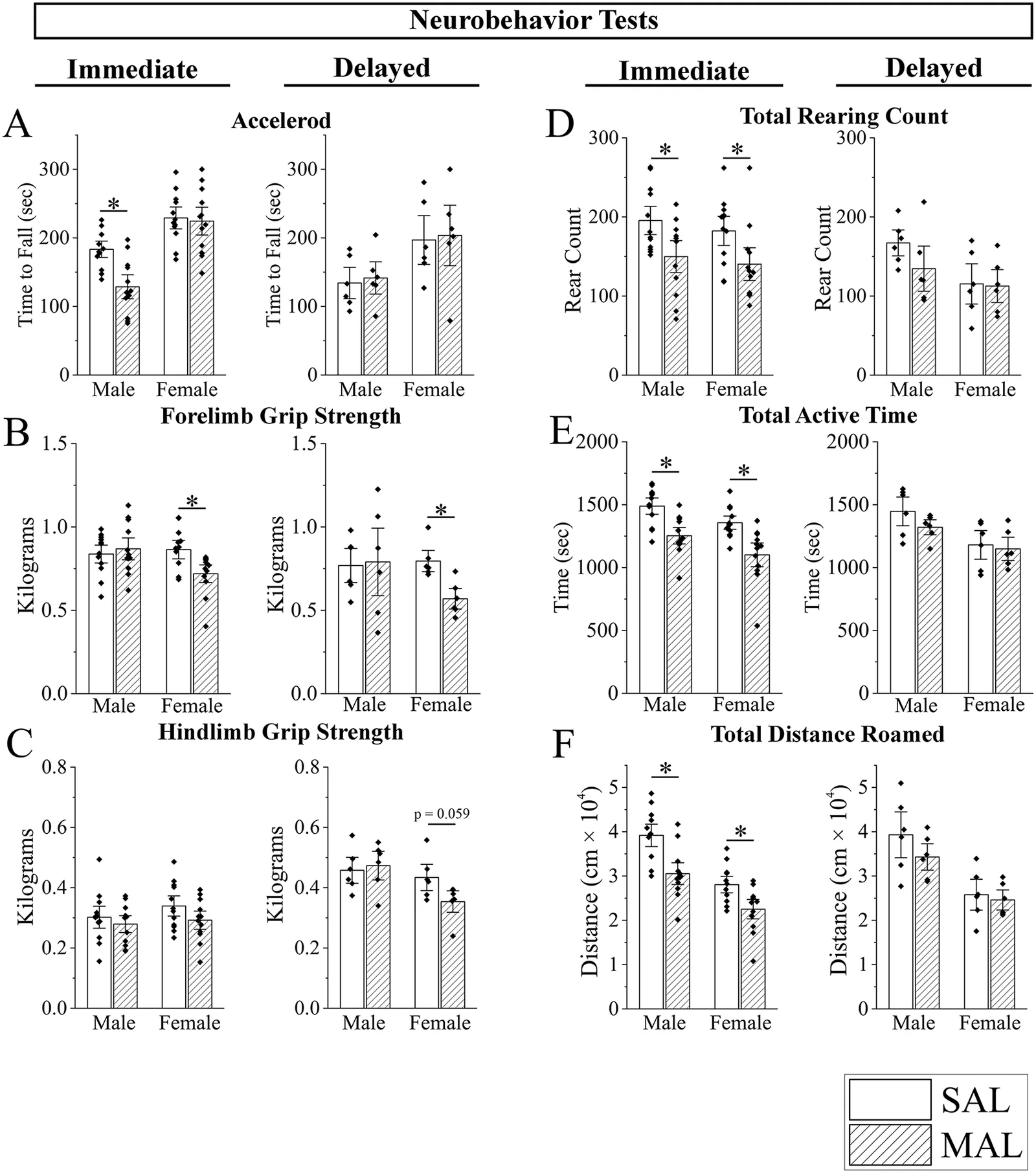
Malathion exposure alters neurobehavioral performance. Bar graphs that demonstrate results from neurobehavioral testing. (A) At the immediate/ acute assessment, male rats exposed to malathion (MAL) exhibited reduced latency to fall on the accelerod compared with saline (SAL) controls (p = 0.0008). No significant differences were observed in females at the immediate time point or in either sex at the delayed time point. (B) Female rats exposed to MAL showed reduced forelimb grip strength compared with saline at both the immediate (p = 0.0098) and delayed (p = 0.003) time point assessments. No significant differences in forelimb grip strength were observed in males. (C) Hindlimb grip strength did not differ between treatment groups at either time point in males or females. (D–F) In the open-field test in males, MAL exposure resulted in significant reductions in total rearing (p = 0.019), active time (p = 0.009), and total distance traveled (p = 0.0013) at the immediate time point. In the open-field test in females, MAL exposure resulted in significant reductions in total rearing (p = 0.033), active time (p = 0.002), and total distance traveled (p = 0.008) at the immediate time point. No detectable differences were found in either males or females following the recovery period. Statistical significance was determined with Student’s t-test. Data are presented as mean ± SEM. Black dots represent individual rats. Immediate n = 12 and delayed n = 6.

The accelerod test was used to evaluate locomotor performance and coordination. At the immediate time point, the MAL-exposed males failed the accelerod approximately 54 seconds (s) earlier than the saline control animals (Fig 1A and Table 1). At the delayed time point, the males were no longer significantly different from controls (Table 1). However, the MAL-exposed females showed no significant differences at either time point (Fig 1A and Table 1). The raw data can be found in S6 Table in S1 File.

**Table 1:** Accelerod and grip strength behavioral outcomes.

	Time points	Saline Male	Malathion Male	Male p-Value (Student’s t-test)	Saline Female	Malathion Female	Female p-Value (Student’s t-test)
Accelerod Latency to failure (s)	Immediate	183.32 ± 27.50	128.68 ± 40.36	0.0008*	229.09 ± 36.78	241.45 ± 55.63	0.790
	Delayed	134.23 ± 37.33	141.55 ± 38.34	0.744	196.85 ± 58.19	203.51 ± 72.01	0.864
Forelimb Grip Strength (kgf)	Immediate	0.84 ± 0.12	0.87 ± 0.15	0.587	0.86 ± 0.13	0.72 ± 0.12	0.0098*
	Delayed	0.77 ± 0.17	0.79 ± 0.33	0.889	0.80 ± 0.10	0.57 ± 0.12	0.003*
Hindlimb Grip Strength (kgf)	Immediate	0.30 ± 0.08	0.28 ± 0.06	0.466	0.34 ± 0.08	0.29 ± 0.07	0.131
	Delayed	0.46 ± 0.07	0.47 ± 0.08	0.726	0.43 ± 0.07	0.35 ± 0.06	0.059

Grip strength is measured as kilograms of force (kgf) generated. Data is presented as mean ± standard deviation whereas n = 12 for immediate time points and n = 6 for delayed. Data passed normality (Shapiro-Wilk) and equal variance (Brown-Forsythe) tests. Statistical significance calculated with Student’s t-test and one-tailed p value is reported. Asterisk denotes significant differences.

Grip strength was used to evaluate general skeletal muscle function (Dataset 1). The males did not present with significant differences in forelimb or hindlimb grip strength, as measured in kilograms of force (kgf), at both the immediate and delayed time points (Fig 1B–1C and Table 1). However, the females presented with a significant decrease in forelimb grip strength at the immediate time point that remained present at the delayed time point (Fig 1B and Table 1). Females did not exhibit significant differences in hindlimb grip strength at either time point, although a strong trend was observed at the delayed time point (Fig 1C and Table 1).

The Open-Field Test was used to assess general locomotor and behavioral activity in rats. Animals were monitored in an open-field arena for 30 min using infrared photobeam breaks in conjunction with video recording. Consecutive photobeam interruptions were used to quantify ambulatory activity, whereas single beam breaks were classified as stereotypic movements, including grooming, scratching, sniffing, and seizure-related behaviors. All measured parameters are provided in Dataset 2, with summary statistics reported in S7 Table in S1 File.

No significant differences were observed between saline and MAL-exposed male or female rats in locomotor speed or stereotypic movements. Similarly, the percentage of time spent in the center of the arena did not differ between groups at either time point, indicating no evidence of anxiety-like behavior (S7 Table in S1 File). At the immediate time point, however, MAL-exposed males and females exhibited a significant ~30% reduction in rearing behavior (Fig 1D and Table 2). This reduction was no longer detected after a 4-week recovery period at the delayed time point. Consistent with reduced exploratory activity, MAL exposure also resulted in an ~ 20% decrease in active time (Fig 1E and Table 2) and a ~ 24% reduction in total distance traveled (Fig 1F and Table 2) at the immediate time point in both sexes. These locomotor effects were not observed following the 4-week recovery period. Together, these findings indicate that MAL exposure produced transient reductions in exploratory motor activity in both male and female rats.

**Table 2:** Open-field motor activity outcomes.

	Time points	Saline Male	Malathion Male	Male p-Value (Student’s t-test)	Saline Female	Malathion Female	Female p-Value (Student’s t-test)
Rearing Count	Immediate	195.50 ± 41.23	149.83 ± 46.64	0.019*	182.23 ± 42.50	140.33 ± 47.83	0.033*
	Delayed	167.17 ± 26.74	134.67 ± 46.57	0.744	115.33 ± 41.53	112.67 ± 34.0	0.906
Total Active Time (s)	Immediate	1488.25 ± 150.38	1253.16 ± 151.06	0.0009*	1356.68 ± 122.15	1101.47 ± 218.27	0.002*
	Delayed	1446.40 ± 186.50	1320.20 ± 97.13	0.172	1180.37 ± 186.31	1148.88 ± 150.41	0.752
Total Distance Roamed (m)	Immediate	391.84 ± 58.46	315.42 ± 56.66	0.0013*	280.60 ± 42.73	225.19 ± 50.67	0.0084*
	Delayed	393.07 ± 84.51	343.06 ± 48.64	0.238	257.89 ± 56.89	246.01 ± 37.00	0.677

Data is presented as mean ± standard deviation whereas n = 12 for immediate time points and n = 6 for delayed. Data passed normality (Shapiro-Wilk) and equal variance (Brown-Forsythe) tests. Statistical significance calculated with Student’s t-test and two- tailed p value is reported. Asterisk denotes significant differences.

### No astrogliosis in the spinal cord following repeated malathion exposure

Glial fibrillary acidic protein (GFAP), a type III intermediate filament protein selectively expressed in astrocytes within the central nervous system, is widely used as a canonical marker of astrocyte activation and astrogliosis. Its upregulation reflects cytoskeletal reorganization, astrocyte hypertrophy, and proliferation that occur in response to neuronal injury, disease, neuroinflammatory signaling, and toxicant exposure, thereby serving as a sensitive indicator of reactive astrocytic responses. Quantification of GFAP immunoreactivity (IR) in lumbar spinal cord segments revealed no evidence of astrocyte activatioAChE activity (nmn following repeated MAL exposure. In single optical confocal micrographs, neither GFAP integrated optical density nor mean GFAP pixel intensity differed between MAL-treated animals and saline controls in either sex at the immediate or delayed time points (S2 Fig). These findings were consistent with the absence of detectable increases in GFAP-IR intensity or astrocyte recruitment, indicating that repeated MAL exposure does not induce astrogliosis in the lumbar spinal cord. Raw data are provided in Dataset 3, with statistical analyses summarized in S8 and S9 Tables in S1 File.

### Lumbar motoneuron soma sizes are altered following repeated exposure to malathion

**Fig 2:**
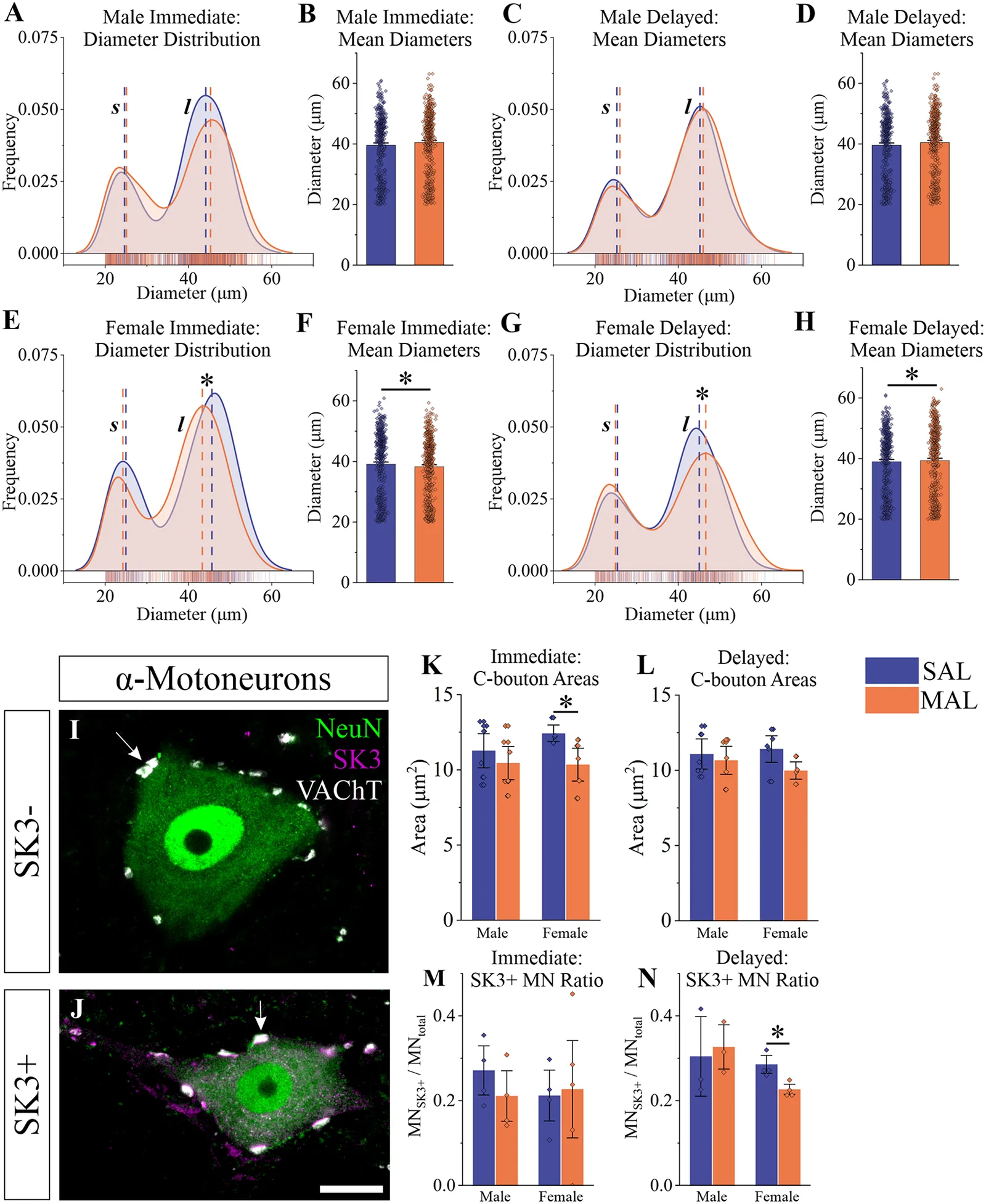
Malathion exposure alters lumbar motoneuron soma size and C-bouton synaptic features. Microscopy images and graphs that are showing results of analysis of motoneuron soma and synaptic structure. (A, C, E, G) Distributions of lumbar motoneuron (MN) soma diameters (>20 µm) exhibit a bimodal pattern. Small (s) and large (l) MN populations were defined by the intersection of best-fit Gaussian curves. Dashed lines indicate the mean diameter for each population. Immediate time point: n = 12 animals; delayed time point: n = 6 animals. Because multiple MNs were sampled per animal, statistical analyses used linear mixed-effects models to account for the nested structure and non-independence of MN measurements within animals. The total number of MNs analyzed per group is reported in Table 3. (B, D, F, H) Mean MN soma diameter across the full distribution for each group. Each dot represents an individual MN. In males, malathion (MAL) exposure did not alter MN diameter immediately after exposure (A, B) or after the recovery period (C, D). In females, MAL reduced the large MN population and decreased overall mean MN diameter at the immediate time point (E, F; p = 0.026 and p = 0.045, respectively). At the delayed time point, the small MN population was unchanged, whereas the large MN population and mean MN diameter were increased relative to saline (SAL) controls (G, H; p = 0.043 and p = 0.012, respectively). (I–J) Representative confocal images of MNs located in ventrolateral lamina IX of the L4–L5 spinal cord. NeuN (green) labels MN somata, SK3 (magenta) labels the SK3 potassium channel isoform, and VAChT (gray) labels cholinergic C-bouton synapses. White arrows indicate C-boutons. (I) SK3-immunoreactive (IR)–negative MN. (J) SK3-IR–positive MN. Scale bar, 20 µm. (K–L) Mean C-bouton area measured immediately after exposure (K) and after recovery (L). Each dot represents the mean value for a single animal. Females exhibited reduced C-bouton area immediately after MAL exposure (p = 0.0245). (M–N) Proportion of SK3-IR MNs measured immediately (M) and after recovery (N). Females showed a significant decrease in the proportion of SK3-IR MNs at the delayed time point (p = 0.006). Data are presented as mean ± SEM.

Motoneuron (MN) size is tightly linked to motor unit recruitment thresholds and intrinsic electrophysiological properties that govern motor output [41–47]. To determine whether repeated MAL exposure alters lumbar MN morphology, MN soma diameters were quantified from single optical confocal sections through the nucleolus of MNs located in lamina IX of the L4–L5 spinal cord (S3 Fig). In total, approximately 4,300 MNs were sampled from 32 rats (16 males and 16 females), and data can be found in Dataset 4. Because multiple neurons were sampled from each animal, statistical inference was performed using mixed-effects models that accounted for clustering within animals rather than treating individual neurons as independent observations. Cells with diameters < 20 μm were excluded as outliers, as these likely represent smaller non-MNs (e.g., interneurons) located near the periphery of lamina IX. Across all experimental groups, MN diameters exhibited a bimodal distribution (Fig 2).

At the immediate time point, MAL exposure did not alter the overall MN diameter distribution in males (Table 3). In contrast, females exhibited a significant 5% reduction in mean MN soma diameter following MAL exposure (Table 3). At the delayed time point, MN diameter remained unchanged in males, whereas females exhibited a significant 6% increase in mean MN diameter following MAL exposure (Table 3).

**Table 3:** Soma diameters of lumbar spinal motoneurons.

	Time points	Saline Male	Malathion Male	Male p-Value	Saline Female	Malathion Female	Female p-Value
Entire Motoneuron Population (µm)	Immediate	39.01 ± 9.75 n = 538	38.67 ± 10.73 n = 615	0.751	37.14 ± 11.88 n = 518	35.20 ± 11.67 n = 636	0.045*
	Delayed	39.59 ± 10.22 n = 419	40.46 ± 10.22 n = 486	0.791	37.01 ± 11.55 n = 498	39.33 ± 11.59 n = 519	0.012*
Small Motoneuron Population (µm)	Immediate	24.64 ± 2.95 n = 142	25.10 ± 3.57 n = 202	0.368	23.31 ± 4.13 n = 197	21.64 ± 4.10 n = 238	0.132
	Delayed	25.19 ± 3.22 n = 117	25.96 ± 3.78 n = 134	0.092	23.52 ± 4.68 n = 186	24.91 ± 3.58 n = 174	0.059
Large Motoneuron Population (µm)	Immediate	44.16 ± 4.94 n = 396	45.31 ± 5.56 n = 413	0.221	45.63 ± 5.24 n = 321	43.32 ± 5.62 n = 398	0.026*
	Delayed	45.16 ± 5.43 n = 302	45.98 ± 5.30 n = 352	0.623	45.05 ± 5.15 n = 312	46.61 ± 6.13 n = 345	0.043*
SK3 Positive Motoneuron Population (µm)	Immediate	42.38 ± 4.43 n = 124	43.40 ± 5.07 n = 102	0.108	42.14 ± 7.01 n = 94	41.79 ± 5.84 n = 142	0.676
	Delayed	43.33 ± 5.89 n = 100	45.09 ± 5.69 n = 124	0.024*	42.59 ± 6.12 n = 126	43.71 ± 8.16 n = 116	0.230
Ratio of SK3 Positive/ Total Motoneurons (Cell Count)	Immediate	0.27 ± 0.07 n = 4	0.21 ± 0.08 n = 3	0.275^†^	0.21 ± 0.08 n = 4	0.28 ± 0.13 n = 4	0.384^†^
	Delayed	0.31 ± 0.11 n = 4	0.33 ± 0.06 n = 3	0.782^†^	0.28 ± 0.02 n = 4	0.22 ± 0.02 n = 4	0.006^†*^

Data are presented as mean ± SD. For cellular measurements, n denotes the number of motoneurons (MNs) analyzed; for ratio data, *n* denotes the number of animals. Because multiple MNs were sampled from individual animals, analyses accounted for the nested data structure using a linear mixed-effects model with experimental group as a fixed effect and animal ID as a random intercept. †Data meeting assumptions of normality (Shapiro–Wilk) and homogeneity of variance (Brown–Forsythe) were analyzed using a two-tailed Student’s *t* test. Asterisks indicate statistically significant differences.

To further examine MN size distributions, populations were subdivided into small and large MN groups based on the intersection of fitted Gaussian curves for each mode. At the immediate time point, cutoff values separating small and large MNs were 31.77 μm (iSAL) and 35.72 μm (iMAL) in males, and 33.35 μm (iSAL) and 31.51 μm (iMAL) in females. In males, MAL exposure produced no significant differences in either the small MN population or the large MN population (Fig 2A and Table 3). In females, however, MAL exposure significantly reduced the diameter of the large MN population by approximately 5%, with no change in the small MN population (Fig 2E and Table 3).

At the delayed time point, cutoff values separating small and large MN populations were 32.92 μm (dSAL) and 34.51 μm (dMAL) in males, and 33.49 μm (dSAL) and 32.86 μm (dMAL) in females. MAL exposure produced trends toward altered diameter in the small MN population in both sexes (Fig 2C and 2G and Table 3). No changes were detected in the large MN population in males (Table 3). In females, however, MAL exposure resulted in an approximately 4% increase in the diameter of the large MN population (Fig 2G and Table 3).

### Alterations in Cholinergic C-boutons and SK3 positive spinal motoneurons following repeated malathion exposure

C-boutons are large cholinergic synapses located on MN somas and proximal dendrites that regulate repetitive firing and MN excitability and are identified by vesicular acetylcholine transporter (VAChT) immunoreactivity (Fig 2I–2J). We examined whether repeated MAL exposure alters the structure of these central cholinergic synapses. *En face* C-bouton area was quantified from single optical confocal sections (Dataset 5). In males, MAL exposure did not alter C-bouton cross-sectional area at either the immediate or delayed time points (Fig 2K–2L and Table 4). In females, MAL exposure produced a significant ~17% reduction in C-bouton area at the immediate time point that partially recovered to a trending ~12% reduction at the delayed assessment (Fig 2K–2L and Table 4). C-bouton synaptic density, measured as the number of VAChT-immunoreactive synapses per MN membrane perimeter sampled in single optical sections, was likewise unchanged by MAL exposure at either time point in either sex (Table 4 and Dataset 6). Together, these findings indicate that repeated MAL exposure produces a modest, female-specific reduction in C-bouton size without altering synapse number, suggesting a form of structural scaling rather than synapse loss. In the broader context of synaptic remodeling, this pattern is consistent with activity-dependent modulation of synaptic efficacy in which cholinergic neuromodulatory inputs undergo subtle structural refinement while preserving overall circuit connectivity [48–52].

**Table 4:** Morphometric analysis of cholinergic C-bouton synapses.

	Time points	Saline Male	Malathion Male	Male p-Value	Saline Female	Malathion Female	Female p-Value
C-bouton Area (µm^2^)	Immediate	11.34 ± 4.48 n = 777, 33, 6	10.49 ± 4.17 n = 752, 29, 6	0.475	12.41 ± 4.00 n = 682, 36, 4	10.36 ± 3.83 n = 905, 44, 5	0.025*
	Delayed	11.10 ± 4.29 n = 573, 24, 5	10.69 ± 4.14 n = 696, 29, 5	0.643	11.63 ± 4.01 n = 807, 43, 5	9.97 ± 3.54 n = 874, 35, 4	0.072
C-bouton Synaptic Density (synapses per µm)	Immediate	0.0461 ± 0.0063 n = 320, 6	0.0509 ± 0.0076 n = 324, 6	0.232	0.0397 ± 0.0070 n = 216, 4	0.0411 ± 0.0050 n = 267, 5	0.737
	Delayed	0.0479 ± 0.0046 n = 269,5	0.0450 ± 0.0095 n = 270, 5	0.529	0.0371 ± 0.0034 n = 215, 4	0.0356 ± 0.0041 n = 213, 4	0.657

Data are presented as mean ± SD. For C-bouton area, n denotes the number of C-boutons, motoneurons (MNs), and animals sampled; for C-bouton density, n denotes the number of MNs and animals sampled, as one density measurement was obtained per MN. C-bouton density was measured in single optical confocal sections and recorded as the number of VAChT-immunoreactive positive synapses per MN perimeter length sampled. Statistical analyses accounted for the nested data structure using linear mixed-effects models with animal as a random effect. p < 0.05; * indicates significant differences.

Because vulnerability differs among motoneuron (MN) subtypes, with MNs innervating slow-twitch muscle exhibiting relative resistance compared with fast-fatigable MNs in motoneuron disease, we examined MNs expressing the small-conductance Ca^2+^ -activated potassium channel (SK3), a marker associated with slow-type MN phenotypes [3,37,53]. Although these MNs are thought to represent a relatively disease-resistant population, it remains unclear whether environmentally relevant neurotoxicant exposures differentially affect this MN subtype.

MAL exposure did not alter SK3-IR ⁺ MN soma diameter (Dataset 7) in either sex at the immediate time point (Table 3). At the delayed time point, SK3-IR ⁺ MNs in males were modestly larger (~5%) in MAL-exposed animals, whereas no size differences were detected in females. The proportion of SK3-IR ⁺ MNs was quantified from micrographs of 60-µm transverse spinal cord sections (Dataset 7). No differences were detected in males at either time point (Fig 2M–2N and Table 3). Females similarly showed no differences immediately after exposure (Fig 2M–2N and Table 3). However, at the delayed time point, females exhibited a significant reduction in the proportion of SK3-IR ⁺ MNs driven by a decrease in the number of SK3-positive cells (Fig 2M–2N and Table 3). Together, these findings show that repeated MAL exposure differentially affects lumbar SK3-IR ⁺ MNs by sex, producing a modest increase in soma size in males and delayed reductions in the proportion of this MN subpopulation in females.

### Decreased neuromuscular junction innervation following repeated malathion exposure

**Fig 3:**
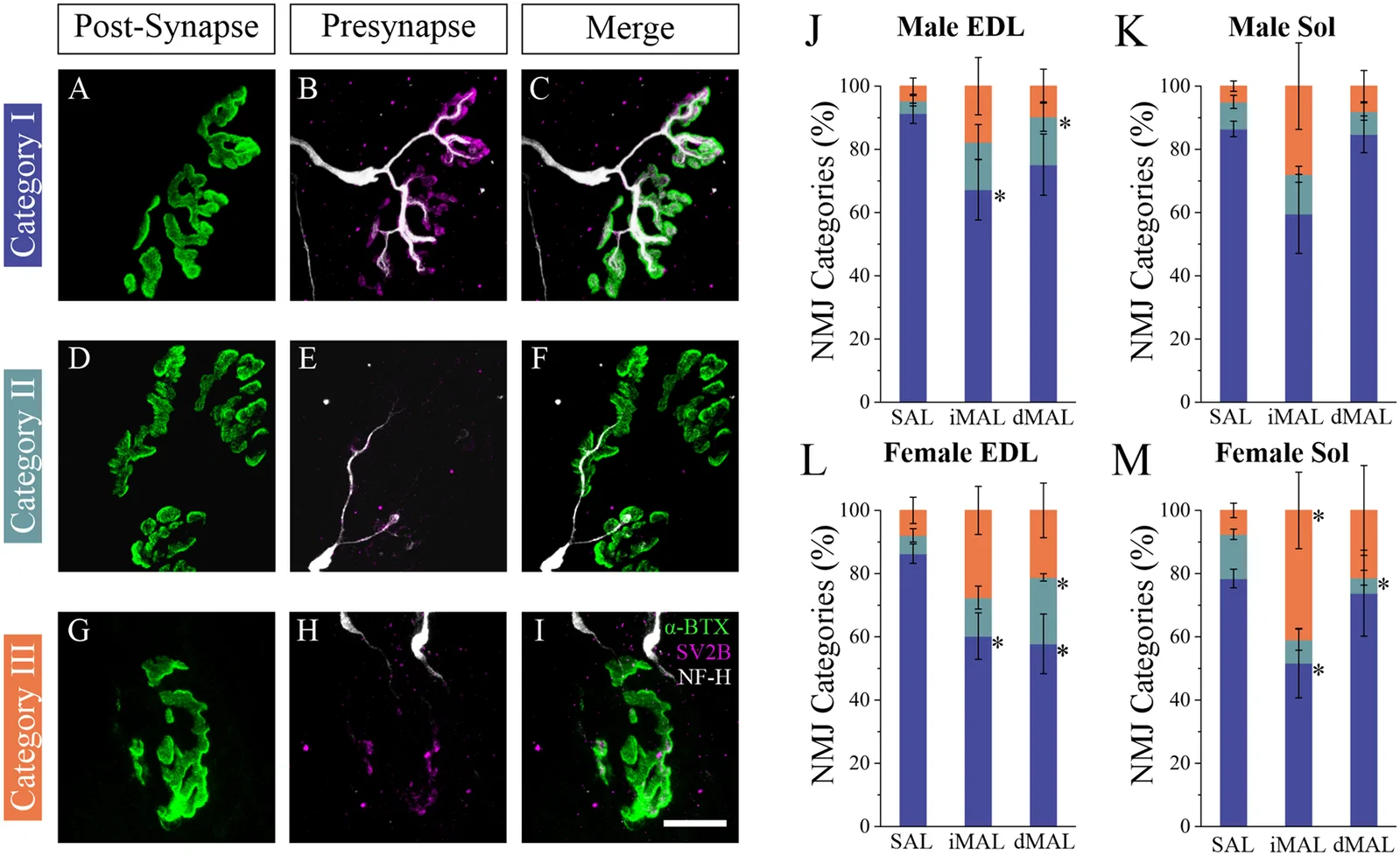
Denervation of neuromuscular junctions following repeated malathion exposure. Microscopy images and related graphs illustrating results of structural innervation analysis. Structural changes in efferent motor axon terminals were assessed at neuromuscular junctions (NMJs) using fluorescent immunohistochemistry and confocal microscopy. Postsynaptic nicotinic acetylcholine receptors (nAChRs; green) were labeled with α-bungarotoxin (αBTX). Presynaptic structures were labeled with antibodies against synaptic vesicle glycoprotein 2B (SV2B; magenta) and neurofilament heavy chain (NF-H; white), marking synaptic vesicles and axonal cytoskeleton, respectively. (A–C) Category I NMJ (fully innervated) showing strong overlap between postsynaptic nAChR clusters (A) and presynaptic markers (B). (D–F) Category II NMJ (partially denervated) showing reduced overlap between postsynaptic (D) and presynaptic (E) structures. (G–I) Category III NMJ (denervated) showing absence of overlap between the nAChR endplate (G) and nerve terminal markers (H). (J–M) Quantification of NMJ categories is shown as stacked bar graphs representing the mean percentage of NMJs in each category. (J) In the male extensor digitorum longus (EDL), Category I NMJs decreased at the immediate time point (P = 0.038), whereas Category II NMJs increased at the delayed time point (P = 0.047) relative to controls. (K) No differences were observed in the male soleus. (L) In the female EDL, Category I NMJs decreased at both the immediate (P = 0.011) and delayed (P = 0.027) time points, whereas Category II NMJs increased at the delayed time point relative to control (P = 0.002) and the immediate time point (P = 0.040). (M) In the female soleus, Category I NMJs decreased (P = 0.031) and Category III NMJs increased (P = 0.016) at the immediate time point, whereas Category II NMJs decreased at the delayed time point relative to control (P = 0.049). Values represent mean ± SEM. Statistical details are provided in Methods and Table 5. Scale bar, 20 μm.

MAL inhibits acetylcholinesterase and disrupts cholinergic neurotransmission. Because the neuromuscular junction (NMJ) is a specialized peripheral cholinergic synapse, we examined whether repeated MAL exposure alters NMJ innervation. NMJs were evaluated using presynaptic neurofilament and postsynaptic α-bungarotoxin labeling and categorized as fully innervated (category I), partially denervated (category II), or fully denervated (category III) (Fig 3A–3I and Table 5). All measured parameters are provided in Dataset 9.

**Table 5:** Neuromuscular junction innervation status across exposure groups.

Sex	Muscle	Category	Saline %	iMAL %	dMAL %	Saline vs iMAL (p)	Saline vs dMAL (p)	iMAL vs dMAL (p)	n (Saline/iMAL/dMAL)
Male	EDL	Cat I	0.91 ± 0.08	0.67 ± 0.24	0.75 ± 0.24	0.038*	0.142	0.573	6/ 6/ 6
Male	EDL	Cat II	0.04 ± 0.04	0.15 ± 0.14	0.15 ± 0.11	0.084	0.047*	0.987	6/ 6/ 6
Male	EDL	Cat III	0.05 ± 0.06	0.18 ± 0.22	0.10 ± 0.13	ANOVA p = 0.642			6/ 6/ 6
Male	Soleus	Cat I	0.87 ± 0.06	0.60 ± 0.30	0.85 ± 0.13	0.062	0.780	0.124	6/ 6/ 5
Male	Soleus	Cat II	0.09 ± 0.05	0.13 ± 0.06	0.07 ± 0.06	ANOVA p = 0.321			6/ 6/ 5
Male	Soleus	Cat III	0.05 ± 0.04	0.28 ± 0.34	0.08 ± 0.11	ANOVA p = 0.164			6/ 6/ 5
Female	EDL	Cat I	0.86 ± 0.07	0.60 ± 0.16	0.58 ± 0.23	0.011*	0.027*	0.848	5/ 5/ 6
Female	EDL	Cat II	0.06 ± 0.05	0.12 ± 0.08	0.21 ± 0.03	0.088^†^	0.002^†^*	0.040^†^	5/ 5/ 6
Female	EDL	Cat III	0.08 ± 0.09	0.28 ± 0.17	0.21 ± 0.21	ANOVA p = 0.212			5/ 5/ 6
Female	Soleus	Cat I	0.79 ± 0.07	0.52 ± 0.25	0.74 ± 0.33	0.031*	0.818	0.252	6/ 5/ 6
Female	Soleus	Cat II	0.14 ± 0.04	0.07 ± 0.07	0.05 ± 0.06	0.157^†^	0.049^†^*	0.483^†^	6/ 5/ 6
Female	Soleus	Cat III	0.08 ± 0.06	0.41 ± 0.27	0.21 ± 0.35	0.016*	0.937	0.333	6/ 5/ 6

*Values are mean ± SD. Category frequencies represent the proportion of neuromuscular junctions classified as fully innervated (Cat I), partially denervated (Cat II), or fully denervated (Cat III). Skeletal muscles examined include the predominately fast type the extensor digitorum longus (EDL) and the predominately slow type soleus. Experimental groups are saline, malathion exposure at the immediate timepoint (iMAL), and malathion exposure at the delayed timepoint (dMAL). Pairwise comparisons were performed using two-tailed t tests or the Holm–Šidák† correction where indicated. In some cases, only overall one-way ANOVA p values are reported without post hoc pairwise comparisons. *Indicates statistical significance.

In male extensor digitorum longus (EDL) muscle, the proportion of fully innervated NMJs was reduced by ~26% at the immediate time point following MAL exposure compared with saline controls, but this difference was not significant at the delayed time point (Fig 3J and Table 5). Category II NMJs showed a corresponding increase, reaching significance at the delayed time point (Fig 3J and Table 5). No significant differences were detected in the male soleus (SOL) muscle, although a trend toward reduced category I NMJs at the immediate time point was observed (Fig 3K and Table 5).

In the female EDL muscle, category I NMJs were significantly reduced at both the immediate and delayed time points (Fig 3L and Table 5). Category II NMJs were significantly increased at the delayed time point relative to the saline and immediate exposure groups. In the female SOL muscle (Fig 3M), category I NMJs were reduced at the delayed time point, accompanied by an increase in category III NMJs (Table 5). Category II NMJs were correspondingly decreased at the delayed time point (Table 5).

Together, these findings indicate that repeated MAL exposure is associated with reduced NMJ innervation and increased partial or complete denervation, particularly in the EDL muscle. These observations are consistent with a transient disruption of NMJ integrity following repeated MAL exposure.

### Morphological alterations of the nicotinic acetylcholine receptors at the neuromuscular junctions

**Fig 4:**
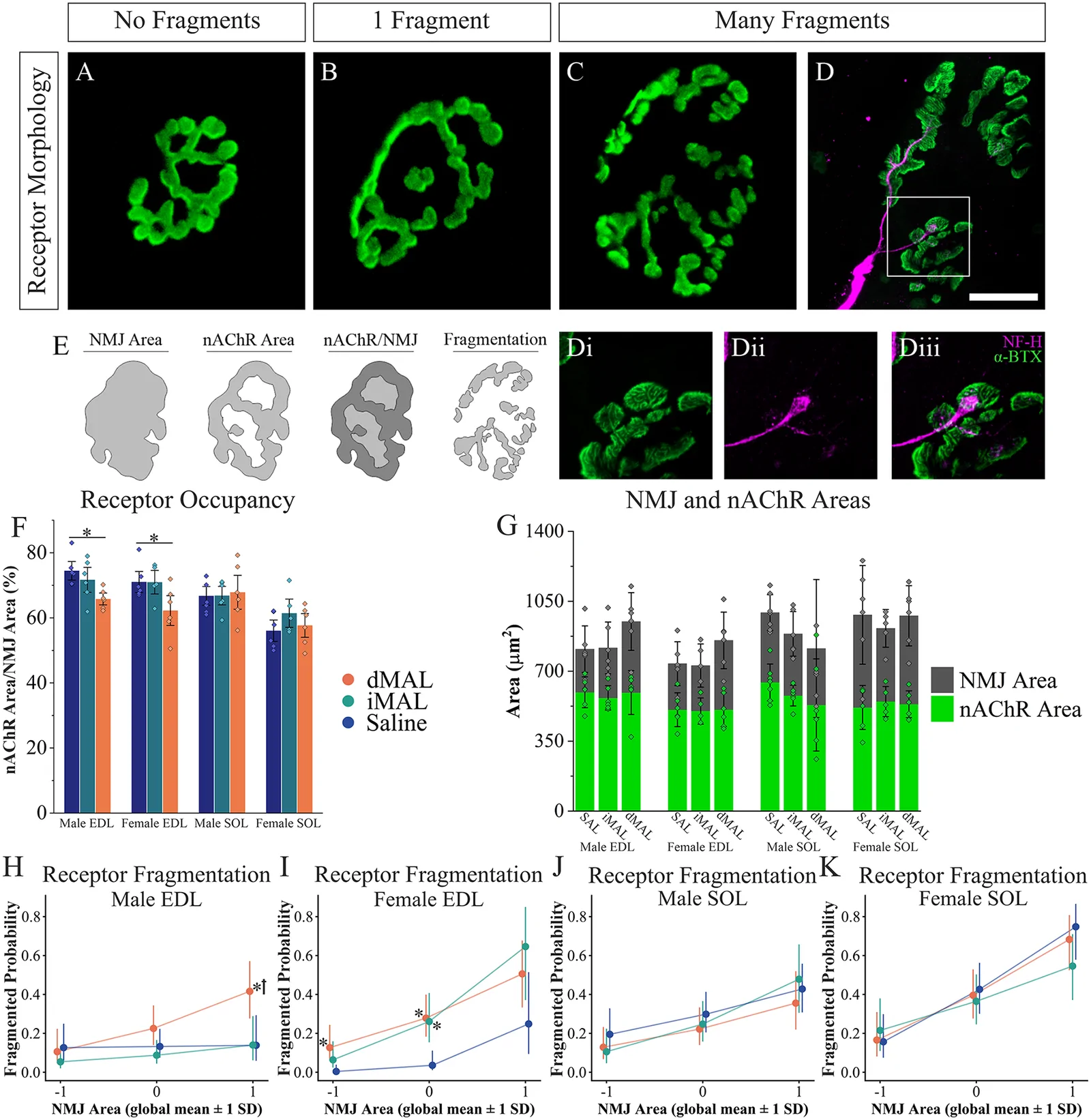
Malathion-induced alterations in postsynaptic nicotinic acetylcholine receptor (nAChR) morphology at the neuromuscular junction. Microscopy images and related graphs showing results from structural analysis of the postsynapse of the neuromuscular junction (NMJ). (A–D) Representative images of nAChR morphology visualized with fluorescent α-bungarotoxin (αBTX; green). (A) Unfragmented NMJ with a continuous nAChR endplate. (B) Single-fragment NMJ containing one discrete region of fragmentation. (C) Multi-fragment NMJ containing multiple discrete nAChR regions within a single endplate boundary. (D) Fragmented NMJ with presynaptic remodeling, showing fragmented nAChR organization and a growth cone–like structure in the presynaptic terminal labeled with neurofilament heavy chain (NF-H; magenta). Scale bar, 20 μm. (Di–Diii) Magnified views of the boxed region in D showing αBTX signal (Di), NF-H signal (Dii), and merged image (Diii). (E) Schematic illustrating quantification metrics. NMJ area was defined by the outer boundary of the nAChR signal; receptor occupancy was calculated as nAChR area divided by NMJ area. Fragmentation was defined as more than one discrete nAChR region within a single NMJ area. (F) Receptor occupancy was reduced in the extensor digitorum longus (EDL) muscle of males (P = 0.003) and females (P = 0.040) at the delayed time point relative to saline controls. (G) Mean NMJ area (gray) and nAChR area (green) did not differ significantly across experimental groups. (H–K) Fragmentation probability plotted against standardized NMJ area (0 = mean [873.21 μm^2^]; ± 1 = ±1 SD [479.46 μm^2^ and 1266.96 μm^2^]). Points represent model-estimated means and error bars indicate 95% confidence intervals. (H) In the male EDL, fragmentation probability increased at +1 SD NMJ area at the delayed time point relative to saline (P = 0.027) and the immediate time point (P = 0.024). (I) In the female EDL, fragmentation probability increased at the delayed time point relative to saline at −1 SD NMJ area (P = 0.011) and at mean NMJ area (P = 0.002), and at the immediate time point relative to saline at mean NMJ area (P = 0.004). (J–K) No fragmentation effects were observed in soleus muscle of either sex. Values are mean ± SD. Sample number and statistical details are listed in Tables 6–7. Asterisk (*) denotes a significant difference detected between SAL and MAL-exposed groups at either time point, and dagger (†) denotes a significant difference detected across iMAL and dMAL.

Repeated subacute exposure to MAL produced subtle alterations in NMJ morphology, visualized by α-bungarotoxin labeling of postsynaptic nicotinic acetylcholine receptors (nAChRs) in flattened confocal image stacks (Fig 4A–4D). NMJ structure was quantified using several morphological parameters, including total NMJ cross-sectional area, postsynaptic nAChR area, receptor occupancy, and fragmentation (Fig 4E and Dataset 10). No differences were observed at the immediate post-exposure time point compared to saline controls across any metric. At the delayed time point, however, receptor occupancy, defined as the percentage of the NMJ area occupied by nAChR fluorescence, was significantly reduced in the EDL of both males and females, decreasing by approximately 8% and 12%, respectively (Fig 4F and Table 6). In contrast, receptor occupancy in soleus muscles did not differ among exposure groups in either sex (Fig 4F and Table 6). Total NMJ cross-sectional area was not significantly altered across exposure groups in either muscle or sex (Fig 4G and Supplemental Table S10 in S1 File), and the absolute area occupied by nAChRs was similarly unchanged (Dataset 10). Together, these results indicate that repeated MAL exposure selectively reduces the proportional coverage of postsynaptic receptors within the NMJ in EDL muscles without producing detectable changes in overall synapse size or total receptor area.

**Table 6:** Nicotinic acetylcholine receptor occupancy within neuromuscular junction area across exposure groups.

Sex	Muscle	Saline	iMAL	dMAL	Saline vs iMAL (p)	Saline vs dMAL (p)	iMAL vs dMAL (p)	n (Saline/ iMAL/ dMAL)
Male	EDL	74.4 ± 4.7	71.7 ± 6.2	65.8 ± 3.0	0.334	0.003	0.100	6/ 6/ 6
Male	Soleus	66.1 ± 4.3	66.3 ± 5.1	68.0 ± 8.3	ANOVA p = 0.842			7/ 6/ 6
Female	EDL	71.0 ± 5.3	71.0 ± 5.4	62.2 ± 7.5	0.978	0.040	0.058	6/ 5/ 6
Female	Soleus	54.6 ± 7.3	61.4 ± 6.5	57.7 ± 5.9	ANOVA p = 0.266			6/ 5/ 6

Values are mean ± SD. Values are % nAChR occupancy which represents the percentage of neuromuscular junction (NMJ) area containing nicotinic acetylcholine receptor signal. Pairwise comparisons were performed using Student’s t tests or Holm–Šidák correction where indicated. In some cases, only overall ANOVA p values are reported without post hoc pairwise comparisons.

Fragmentation of the nAChR endplate was frequently observed during analysis (Fig 4C–4D), prompting quantification of its prevalence. Fragmentation was defined as a binary outcome: NMJs exhibiting a single continuous nAChR region were classified as unfragmented, whereas those containing more than one discrete nAChR region were classified as fragmented (Fig 4E and Dataset 10). In the EDL, both sexes exhibited an increased proportion of fragmented NMJs across treatment conditions (Table 7), whereas soleus muscles showed no significant differences among groups (Table 7).

**Table 7:** Neuromuscular junction fragmentation across exposure groups.

Sex	Muscle	Saline	iMAL	dMAL	Saline vs iMAL (p)	Saline vs dMAL (p)	iMAL vs dMAL (p)	n (Saline/ iMAL/ dMAL)
Male	EDL	0.13 ± 0.09	0.09 ± 0.14	0.28 ± 0.14	0.310	0.054	0.039*	6/ 6/ 6
Male	Soleus	0.35 ± 0.15	0.28 ± 0.14	0.22 ± 0.17	ANOVA p = 0.364			7/ 6/ 6
Female	EDL	0.05 ± 0.04	0.21 ± 0.16	0.29 ± 0.20	0.082	0.034*	0.485	6/ 5/ 6
Female	Soleus	0.44 ± 0.15	0.37 ± 0.15	0.47 ± 0.16	ANOVA p = 0.581			6/ 5/ 6

Values are mean ± SD. Percent Fragmentation represents the proportion of neuromuscular junction (NMJ) area exhibiting discontinuous acetylcholine receptor clusters. Pairwise comparisons were performed where indicated. In some cases, only overall ANOVA p values are reported without post hoc pairwise comparisons when not significant. Significant is indicated with asterisk.

To assess determinants of fragmentation probability, we applied a logistic mixed-effects model with NMJ area, treatment group, muscle, and sex as predictors. The model was fit to >1,400 NMJs. Simple slopes describing the relationship between NMJ area and fragmentation probability were estimated for each Group × Muscle × Sex combination; 10 of 12 strata showed significantly positive slopes (Fig 4H–4K and S11 Table in S1 File), indicating that fragmentation probability generally increased with NMJ size.

Model-based comparisons at standardized NMJ areas revealed sex-specific treatment effects in the EDL. In males, fragmentation probability was increased only in large NMJs (+1 SD) at the delayed time point (Fig 4H; saline = 13.9 ± 5.8% vs. dMAL = 41.6 ± 7.7%, P = 0.027, Wald z-test). Consistent with this pattern, the NMJ size–dependent fragmentation slope was significantly increased in the male EDL at the delayed time point, as indicated by a significant NMJ Area × dMAL × Male interaction (log-odds = 2.05 ± 0.84, P = 0.014; S11 Table in S1 File).

In contrast, the female EDL showed increased fragmentation probability at the delayed time point in small (−1 SD) and average-sized NMJs (0 SD) (Fig 4I; −1 SD: saline = 0.4 ± 0.5% vs. dMAL = 12.7 ± 4.5%, P = 0.011; 0 SD: saline = 3.6 ± 2.1% vs. dMAL = 27.8 ± 5.6%, P = 0.002; Wald z-tests). Additionally, fragmentation probability was increased at average NMJ size at the immediate time point relative to saline (saline = 3.6 ± 2.1% vs. iMAL = 27.8 ± 5.6%, P = 0.004). The relationship between fragmentation and NMJ area in the female EDL at the delayed time point showed a nonsignificant trend toward a flattened slope, reflected by the NMJ Area × dMAL interaction term (log-odds = −1.21 ± 0.73, P = 0.098; S11 Table in S1 File). No treatment effects were detected in soleus muscles of either sex.

### Galantamine treatment protects against the effects of repeated malathion exposure

To determine whether pharmacological protection of AChE could mitigate the effects of repeated MAL exposure, we administered galantamine (GAL), a reversible AChE inhibitor, as a prophylactic treatment. Reversible inhibitors transiently occupy AChE binding sites and can limit access to irreversible organophosphate inhibitors such as MAL, providing a strategy to evaluate whether observed effects depend on cholinesterase inhibition. GAL-only control and MAL + GAL treatment groups were exposed in parallel with the saline and MAL experimental groups.

GAL treatment was well tolerated. No overt cholinergic symptoms were observed in treated animals, and plasma AChE activity did not differ significantly among groups at either time point in males or females (Table 8). These findings indicate that GAL administration did not measurably alter systemic cholinesterase activity under the exposure conditions used and therefore enabled evaluation of its potential protective effects against repeated MAL exposure.

**Table 8:** Biochemical and behavioral outcomes following galantamine treatment.

Measure	Sex	Timepoint	Saline	MAL	GAL	MAL + GAL	p-value (ANOVA)	n (Saline/ MAL/ GAL/ MAL + GAL)
AChE activity (nmol·min^-1^·mL^-1^)	Male	Immediate	5.26 ± 2.30	5.75 ± 1.05	4.10 ± 1.98	3.63 ± 1.80	0.220	6/ 6/ 6/ 5
AChE activity (nmol·min^-1^·mL^-1^)	Male	Delayed	4.37 ± 1.66	4.68 ± 2.03	4.54 ± 2.41	4.42 ± 2.19	0.994	5/ 6/ 6/ 5
AChE activity (nmol·min^-1^·mL^-1^)	Female	Immediate	5.98 ± 1.79	8.25 ± 2.29	9.33 ± 3.42	6.83 ± 1.59	0.104	6/ 6/ 6/ 6
AChE activity (nmol·min^-1^·mL^-1^)	Female	Delayed	8.35 ± 1.45	8.81 ± 1.58	8.25 ± 1.82	6.44 ± 1.95	0.115	5/ 6/ 6/ 5
Accelerod latency to fall (s)	Male	Immediate	183.32 ± 27.50	128.68 ± 40.36	157.93 ± 30.87	164.00 ± 38.50	Saline vs. MAL p = 0.004 (Holm-Sidak)	12/ 12/ 12/ 12
Accelerod latency to fall (s)	Male	Delayed	134.23 ± 37.33	141.55 ± 38.34	176.93 ± 26.66	159.77 ± 39.38	p = 0.195	6/ 6/ 6/ 6
Accelerod latency to fall (s)	Female	Immediate	229.09 ± 36.78	224.45 ± 46.91	209.01 ± 60.48	241.45 ± 55.63	p = 0.478	12/ 12/ 12/ 12
Accelerod latency to fall (s)	Female	Delayed	196.85 ± 58.19	203.51 ± 72.01	164.65 ± 72.01	233.93 ± 49.79	p = 0.333	6/ 6/ 6/ 6
Forelimb grip strength (kgf)	Male	Immediate	0.84 ± 0.12	0.87 ± 0.15	0.83 ± 0.18	0.86 ± 0.17	p = 0.932	12/ 12/ 12/ 12
Forelimb grip strength (kgf)	Female	Immediate	0.86 ± 0.13	0.72 ± 0.12	0.88 ± 0.14	0.87 ± 0.15	GAL vs. MAL p = 0.037 MAL + GAL vs. MAL p = 0.046 Saline vs. MAL p = 0.047 (Holm-Sidak)	12/ 12/ 12/ 12

Values are mean ± SD. MAL = malathion exposure; GAL = galantamine treatment. Behavioral outcomes include accelerod latency to fall and forelimb grip strength. Motoneuron measures include C-bouton density and glial fibrillary acidic protein (GFAP) immunofluorescence intensity and integrated optical density. Sample sizes are shown as n (Saline/MAL/GAL/MAL + GAL). Statistical comparisons were performed using ANOVA with Holm–Šidák post hoc tests where appropriate. Saline and MAL groups were previously reported in the main manuscript and are reproduced here to permit direct statistical comparison with galantamine-treated groups. Statistical analyses were performed across all groups within the same model.

**Fig 5:**
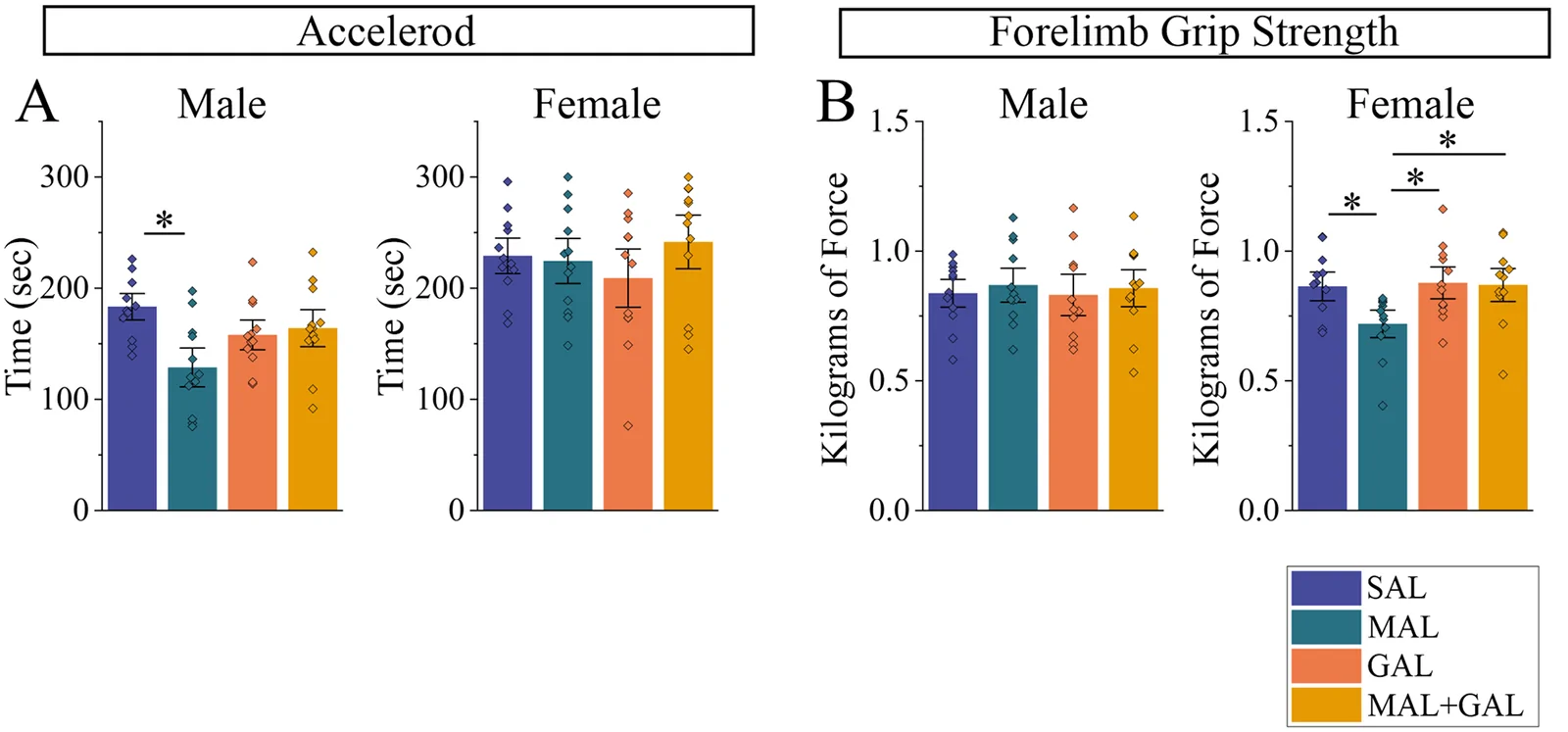
Galantamine attenuates malathion-induced neurobehavioral deficits. Neurobehavioral outcomes measured at the immediate time point for saline (SAL), malathion (MAL), galantamine (GAL), and MAL + GAL groups. (A) Accelerod latency to fall. MAL-exposed males exhibited reduced latency to fall relative to SAL controls (P = 0.004, Holm–Šidák post hoc comparison), whereas GAL-treated groups did not differ from SAL controls. No differences were observed among female groups (P = 0.478, one-way ANOVA). (B) Forelimb grip strength. Grip strength did not differ among male groups (P = 0.932, one-way ANOVA). In females, MAL exposure reduced grip strength compared with SAL, GAL, and MAL + GAL groups (SAL vs. MAL, P = 0.047; MAL vs. GAL, P = 0.037; MAL vs. MAL + GAL, P = 0.046). SAL and MAL groups were previously reported in the main manuscript and are reproduced here to permit statistical comparison with GAL-treated groups. Statistical analyses were performed across all groups within the same model. Error bars represent SEM; n = 12 per group.

Consistent with this interpretation, GAL-only animals were largely indistinguishable from saline controls across most measures. GAL treatment did not alter motor performance on the accelerod (Fig 5A and Table 8) or forelimb and hindlimb grip strength (Fig 5B, Table 8 and Dataset 1). Similarly, no differences were detected in soma size in GAL-exposed animals across the entire MN population, but there was an approximately 5% decrease in the soma size of large MNs in GAL-exposed animals (Dataset 4 and S12 Table in S1 File). Measures of C-bouton structure were also unchanged, with no differences in average C-bouton area or density on MNs in GAL controls of either sex at either time point (Datasets 5–6, and S15–S16 Tables in S1 File). In addition, GAL exposure did not affect SK3 + MN diameters or the SK3 + /MN ratio (Datasets 7–8 and S13–S14 Tables in S1 File), nor did it induce spinal cord astrogliosis (Dataset 3 and S17–S18 Tables in S1 File). A small number of transient behavioral differences were observed. Specifically, GAL-exposed animals demonstrated increased open-field activity at the immediate time point, including ambulatory beam breaks, and active time, however, these differences were not present at the delayed time point (Dataset 2 and S7 Table in S1 File).

Having established that GAL alone produced minimal biological effects, we next assessed its ability to protect against MAL-induced deficits. When GAL was administered ~30 min prior to MAL exposure, protective effects were observed across multiple behavioral outcomes (Fig 5). In males, GAL prevented the decline in accelerod performance observed in MAL-only animals at the immediate time point (Fig 5A and Table 8). Similarly, in females, MAL + GAL animals did not exhibit the reduction in forelimb grip strength detected in the MAL-only group at the immediate assessment (Fig 5B and Table 8). In addition, MAL + GAL treatment prevented the open-field motor activity deficits observed in MAL-exposed animals in both sexes (Dataset 2 and S7 Table in S1 File).

Not all outcomes were protective. At the immediate assessment, average MN diameter was significantly increased in the MAL + GAL group relative to all other groups (Dataset 4 and S12 Table in S1 File). This effect persisted after the 4-week recovery period, with MN diameter remaining elevated in MAL + GAL animals relative to all groups except MAL, which showed a near-significant difference (Dataset 4 and S12 Table in S1 File).

## Discussion

Organophosphates (OPs) are neurotoxicants and include commercially available pesticides, as well as certain chemical nerve agents, which can disrupt motor performance in humans and animals [54]. The primary mechanism of action for OPs is AChE inhibition. There is also evidence that repeated subacute OP exposures, which are more common but do not severely impact AChE activity (<10%), are linked to adverse neurological effects in humans and animals [18–20,27,55,56]. Furthermore, multiple neurodegenerative diseases appear to be associated with chronic subacute exposure to OPs [57,58]. In this study, we examined the effects of repeated “occupational-like” subacute exposure to MAL, a commonly used OP pesticide, in a rat model. Following the 20 daily exposures to MAL, the rats appeared in good health, with no symptoms of cholinergic distress or crisis, and no significant impacts on plasma cholinesterase activity. Despite this, we found changes in locomotor behavior with structural alterations in the motor unit. This highlights a need to better understand the impacts of occupational or repeated subacute OP exposure at levels that do not alter blood cholinesterase activity and to identify potential health consequences.

In both humans and rodents, several blood esterases are expressed, including AChE, butyrylcholinesterase, carboxylesterase, and paraoxonase, all of which are sensitive to MAL [59–61]. Clinically, the confirmation of acute OP poisoning may include measurements of blood cholinesterase activity, with greater than 90% inhibition of activity associated with severe toxicity [62]. Unfortunately,subacute exposures that do not produce clinical cholinergic symptoms and inhibit blood cholinesterase activity by less than 20% may not be detected in humans due to wide interpersonal variability. Thus, unless a pre-exposure baseline is established in each individual, measuring blood cholinesterase activity levels may not be informative for subacute occupational exposures. In rodent OP studies, there is a precedent to measure plasma AChE activity levels due to a general correlation with brain AChE activity levels. However, there is evidence that suggests MAL may have a larger impact on plasma cholinesterase activity than brain AChE activity levels [20,63]. In this study, a commercially available AChE kit was used to monitor plasma cholinesterase activity; however, it is likely that these kits are detecting other cholinesterase activities as well. Our results are consistent with other studies that showed repeated exposure to <50 mg/kg MAL did not impact blood cholinesterase activity [20,64,65]. It is unknown if the health effects reported here and elsewhere are due to a moderate but transient impact on cholinesterase activity or some other mechanism.

In this study, the administration of GAL, a competitive and reversible AChE inhibitor, was protective against repeated exposure to MAL. Reversible AChE inhibitors are generally used therapeutically in conditions such as myasthenia gravis, dementia, and Alzheimer’s disease. In addition, this class of drugs can also be used prophylactically to protect against OP nerve agents, such as sarin, which, like MAL, are irreversible inhibitors of AChE activity. GAL requires a lower concentration to reversibly inhibit 50% AChE enzymatic activity (IC_50_) compared to irreversible MAL (IC_50_ = 560 nM and 160–210 µM, respectively). GAL has been shown to inhibit both AChE and butyrylcholinesterase activity in rodents and humans [66–68]. Given that we did not find significant decreases in blood cholinesterase activity levels with either MAL exposure or GAL treatment, we were surprised by the effectiveness of GAL. This leads us to speculate that either 1) a small inhibition of cholinesterase activity is biologically sufficient to cause health effects, 2) there are additional mechanisms involving cholinesterase activities that may contribute to health effects beyond traditional roles, or 3) there are compensatory or homeostatic effects on blood cholinesterase activity that require further exploration. Regardless, this highlights the need for additional studies to determine the cellular mechanisms involved in repeated subacute OP exposures to optimize therapeutic strategies.

### Repeated malathion exposures effects motor function

Previous human studies have reported neurobehavioral impairments following exposure to MAL, including reduced motor speed and coordination, which have been interpreted as evidence of disrupted sensorimotor integration [56]. Sensorimotor integration depends on coordinated processing across multiple neural substrates, including the cerebral cortex, thalamus, basal ganglia, cerebellum, spinal cord, and peripheral musculature, and is essential for the execution of precise and adaptive motor behaviors. Within this framework, the observed impairments in the accelerod task suggest that MAL exposure compromises the integration of motor planning and execution processes required to maintain balance and adjust locomotor output under increasing task demands. Importantly, the preservation of performance in MAL-exposed animals treated with GAL supports the interpretation that these deficits are, at least in part, mediated by cholinergic dysfunction and are amenable to pharmacological intervention.

Consistent with this interpretation, alterations in open-field behavior further indicate that MAL exposure disrupts motor function rather than inducing anxiety-like phenotypes. Although open-field paradigms are commonly used to dissociate locomotor and affective behaviors, the absence of changes in center time, despite reductions in total distance traveled, active time, and rearing, argues against a primary effect on anxiety. Instead, these findings point toward deficits in motor output or neuromuscular performance. The concomitant reduction in grip strength observed in female rats strengthens this interpretation, suggesting that both central motor control and peripheral neuromuscular function may be affected. Notably, the recovery of locomotor activity following a four-week washout period indicates that these impairments are reversible, which may reflect compensatory plasticity or the resolution of transient neurochemical disruptions rather than permanent structural damage.

The present findings align with a broader, albeit heterogeneous, literature examining the effects of repeated low-level MAL exposure in rodents. While some studies have reported no measurable deficits in locomotor activity at moderate exposure levels (e.g., 150 mg/kg) [69], others have identified impairments only at higher doses (>250 mg/kg) [70,71]. Such discrepancies likely reflect methodological variability, including differences in exposure paradigms (e.g., route, duration), timing of behavioral assessments, and sensitivity of the behavioral assays employed. In this context, the deficits observed here following occupationally relevant exposure levels suggest that subtle impairments in motor coordination and activity may emerge under conditions that more closely model subchronic or chronic low-dose exposure. Moreover, these results are consistent with reports from other organophosphate pesticide studies, which similarly demonstrate disruptions in motor function and coordination even in the absence of overt toxicity [18,39,72,73].

### Repeated malathion exposure does not cause spinal astrogliosis

In toxic exposures, injury, and many diseases of the central nervous system, astrocytes can become reactive, quantified by measurements of increased GFAP immunoreactivity, and undergo morphological changes and/or proliferation [74]. Astrogliosis has been reported in rodent models following repeated exposure to MAL. However, reports vary depending on factors such as exposure concentration, co-exposure conditions, route of exposure, and the anatomical location examined. For example, dos Santos and colleagues identified astrogliosis in the hippocampus of rodents following repeated subcutaneous MAL exposure (30 mg/kg and 100 mg/kg for 15 days) [20]. It has also been shown that co-exposure of MAL and radiation produces a synergistic effect on astrogliosis in the rodent hippocampus, highlighting the need to consider the impact of co-exposures with other environmental stressors in toxicological assessments [75]. To our knowledge, we are the first to examine evidence of astrogliosis in the rodent spinal cord following repeated low-dose MAL exposures. Despite astrogliosis being reported in the brain, we did not find evidence of astrogliosis in the spinal cord based on GFAP immunoreactivity. These observations suggest that spinal inflammation is unlikely to significantly contribute to MAL-induced changes in MN structure, NMJ structure, or neurobehavioral function.

### Repeated exposure to malathion causes sex-dependent impacts on motoneuron soma size

MN soma size is closely associated with motor unit phenotype, with smaller MNs typically innervating slow, fatigue-resistant units and larger MNs innervating fast, more fatigable units [76,77]. In accordance with the size principle, MN size is a key determinant of recruitment threshold, excitability, and motor output [37,42–46,76–79]. Within this framework, the absence of size changes in males suggests relative resistance to structural remodeling following MAL exposure. In contrast, females exhibited pronounced and bidirectional plasticity, characterized by an initial reduction in MN size, most evident in large MNs, followed by hypertrophy of this population after recovery. These findings indicate sex-dependent and subtype-specific remodeling, with large MNs showing the greatest susceptibility.

To further refine motor unit identity, we assessed expression of small conductance calcium-activated potassium channel isoform 3 (SK3), a marker associated with a subset of slow-type MNs [37]. While males again showed no detectable changes, females exhibited a reduced proportion of SK3-positive MNs alongside a trend toward increased size in this population after recovery. This pattern is consistent with either selective vulnerability of this population of slow-type MNs or a shift toward faster phenotypic characteristics, although distinguishing between these possibilities will require lineage-resolved approaches.

The basis for these sex differences remains unclear but may reflect differences in metabolism of MAL or its active metabolite, malaoxon, or in downstream pathways such as cholinergic signaling, calcium handling, or oxidative stress. Notably, the most robust effects were observed in large MNs, whereas smaller and SK3-positive MNs were comparatively spared, suggesting that vulnerability may scale with MN size or associated metabolic demands. One possible explanation for our data is that sex hormones differentially modulate cholinergic signaling, oxidative stress responses, or calcium homeostasis in MNs, such that females exhibit greater short-term structural plasticity whereas males may experience reduced long-term resilience to cumulative stressors; however, this hypothesis requires direct experimental testing.

Alterations in MN soma size are a recognized feature of neurodegenerative disease. In amyotrophic lateral sclerosis, larger MNs are preferentially vulnerable [80–83], and early hypertrophy has been reported prior to degeneration [84,85]. Similar hypertrophic responses have been reported in other models of MN disease, including the Wobbler mouse [86,87] and Spinal Muscular Atrophy (SMA) mouse models [88]. Neuronal hypertrophy is also observed in other neurodegenerative contexts, such as early Alzheimer’s disease, where it often precedes subsequent atrophy and cell loss [89–92]. To our knowledge, the present findings provide the first evidence that exposure to an environmental toxicant such as MAL can induce lumbar MN soma size alterations that parallel those reported in neurodegenerative disease models.

Mechanistically, MN hypertrophy has been proposed to reflect a compensatory response to altered excitability, metabolic stress, or disrupted calcium homeostasis [93]. Consistent with this, early hyperexcitability is reported in several MN disease models, and computational studies suggest that increases in soma size may help stabilize firing properties under stress [84,88,93–100]. In this context, the MAL-induced changes observed here may represent adaptive remodeling aimed at preserving motor function. However, whether such changes are ultimately protective or contribute to long-term vulnerability remains unresolved and will require longitudinal functional and anatomical analyses.

Given that MN soma size is indicative of MN phenotype (fast- vs. slow-twitch, fatigable vs. fatigue resistant), large numbers of neurons were sampled from each animal to accurately characterize the distribution of cell sizes along a continuum within each animal. However, statistical inference was performed using hierarchical mixed-effects and GEE models in which the individual animal, rather than individual MNs, served as the experimental unit, thereby accounting for within-animal correlation while avoiding pseudoreplication. Thus, a limitation of this study is the relatively modest number of animals within each sex and treatment group, which may have reduced sensitivity for detecting subtle treatment effects in the presence of normal biological variability. Future studies including larger animal cohorts will be valuable to further refine effect size estimates and assess the reproducibility of these findings.

### Repeated malathion exposures cause structural changes at the Neuromuscular Junction

The NMJ is the terminal cholinergic synapse of spinal MNs. Therefore, the MN alterations observed following MAL exposure prompted the investigation of potential MAL-induced morphological changes at the NMJ. NMJs from the extensor digitorum longus (EDL) and soleus (SOL) muscles were used for two primary reasons. First, both hindlimb muscles are innervated by lower lumbar spinal MNs. Second, they provide a physiologically relevant comparison between predominately fast- and slow-twitch muscle groups, where ~95% of EDL fibers are fast twitch and ~90% of SOL fibers are slow-twitch [101].

In almost all muscles and sexes, MAL exposure significantly reduced the proportion of fully innervated NMJs relative to the saline controls, except for the male SOL, which showed a strong trend. These findings indicate that low-level, repeated MAL exposures are sufficient to induce features consistent with peripheral neuropathy. Because myofiber denervation is known to elicit muscle atrophy and weakness [102,103], the neurobehavioral deficits observed in this study may partially reflect NMJ dysfunction. However, the threshold of denervation required to produce overt muscle weakness is unknown. Interestingly, both fast- and slow-twitch fibers exhibited similar denervation patterns following MAL exposure.

Postsynaptic nicotinic receptors (nAChRs) displayed several morphological alterations after repeated MAL exposure. NMJs associated with fast-twitch myofibers showed decreased receptor occupancy but increased fragmentation prevalence at the delayed time point, a pattern consistent with active remodeling during recovery [104,105]. However, NMJs on the slow-twitch fibers did not follow this pattern. In males, the SOL trended toward reduced NMJ area, whereas females showed no trends in morphological changes. Importantly, SOL muscles in both sexes had a higher proportion of fragmented NMJs, even in saline control conditions and across all NMJ areas. Thus, increases in SOL NMJ fragmentation should be interpreted cautiously. A plausible explanation could be that NMJs associated with slow-twitch fibers are intrinsically more prone to structural fragmentation compared to fast-twitch NMJs, but further testing is required for confirmation.

**Fig 6:**
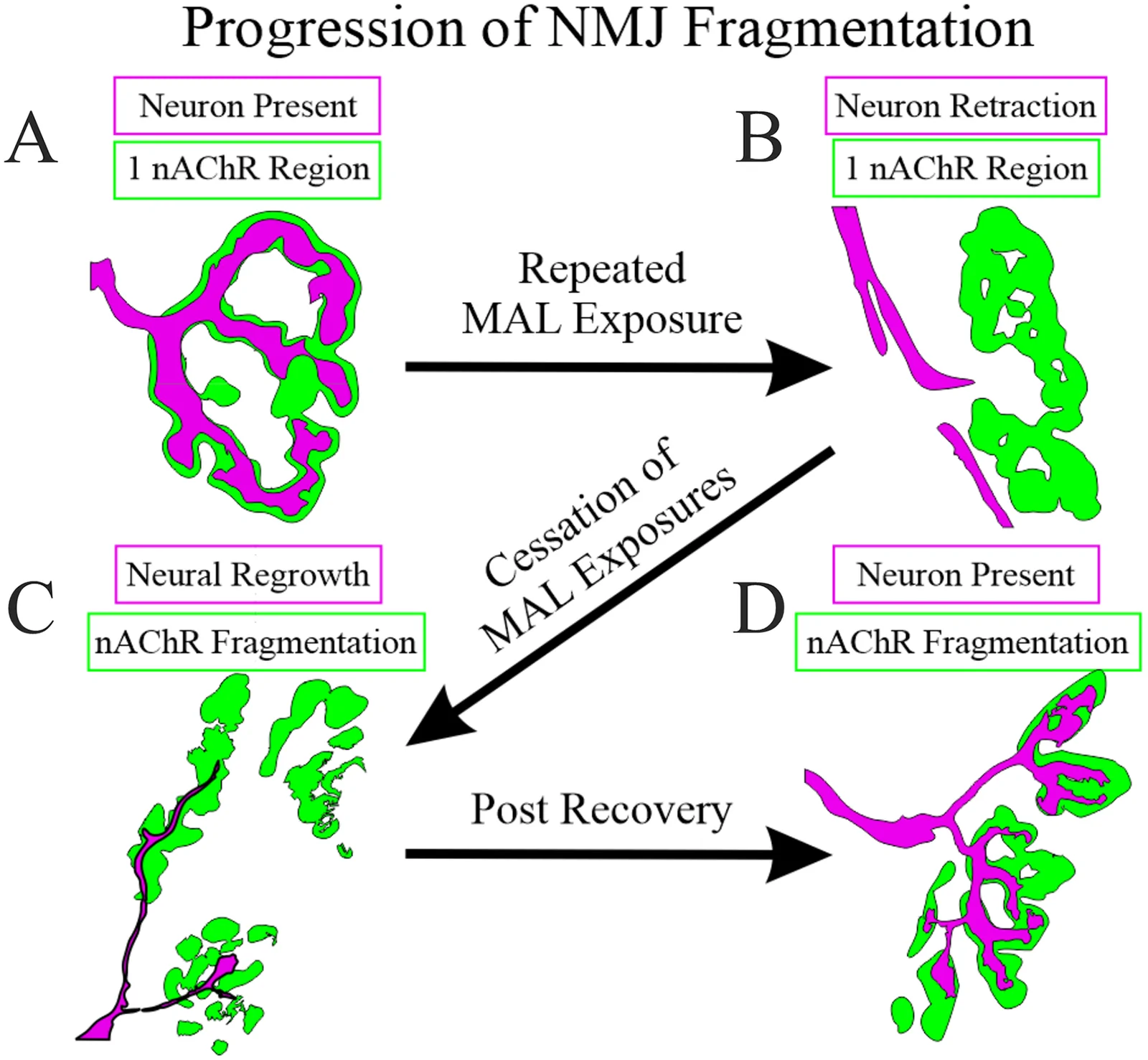
Proposed model of neuromuscular junction structural progression following repeated malathion exposure. Schematic NMJ structures illustrate a hypothesized sequence of synaptic remodeling based on phenotypes observed in fluorescence microscopy images. Each model NMJ was derived from representative images of motor endplates labeled for nicotinic acetylcholine receptors (nAChRs; green) and motor axon terminals (magenta). (A) Pre-exposure phenotype, characterized by an intact motor nerve terminal closely aligned with a continuous nAChR endplate. (B) Early denervation phenotype, showing retraction of the motor axon terminal and reduced overlap with the nAChR region while the postsynaptic endplate remains largely contiguous. This morphology was most frequently observed at the immediate time point following malathion exposure. (C) Reinnervation phase, in which the motor axon terminal reestablishes contact with the associated endplate during a putative reparative reinnervation process. (D) Post-reinnervation phenotype, depicting a reinnervated but structurally fragmented NMJ, consistent with remodeling following repeated cycles of denervation and reinnervation. This model summarizes a proposed trajectory of NMJ structural alterations induced by repeated malathion exposure.

Age-related studies have shown that NMJs become increasingly fragmented over time [106–108]. Such fragmentation has been linked to physiological degeneration and regeneration cycles in myofibers [106], as well as pathological denervation/reinnervation events [107,109,110]. Postsynaptic currents examined in fragmented NMJs from aging mice showed that synaptic transmission is preserved, suggesting functional fidelity despite morphological fragmentation [108]. Thus, the elevated fragmentation observed in EDL NMJs after the 4-week recovery period likely reflects regenerative remodeling following prior denervation (Fig 6). Supporting this interpretation, the male EDL muscles showed partial recovery of innervation that coincided with increased fragmentation. On the other hand, the female EDL did not show a clear reinnervation response after the recovery period. This divergence may be related to the increased MN diameter observed in females but not in males. Additionally, we detected a sex-specific alteration in the size-dependent fragmentation slope across NMJ area. A significantly steeper fragmentation slope was observed in male EDL at the delayed time point, indicating that fragmentation probability increased preferentially in larger NMJs. In contrast, females exhibited a non-significant trend toward a reduced size-dependent slope at the delayed time point, suggesting that fragmentation probability may be uniformly increased across NMJ sizes rather than preferentially increasing with larger NMJs. One possible explanation is sex-dependent toxicokinetics of MAL or malaoxon whereby prolonged retention in females could delay reinnervation dynamics or modify the postsynaptic remodeling process.

## Conclusions

Neurodegenerative diseases are underpinned by a progressive loss of neurological structures and function, and examples include dementia, Parkinson’s disease, Alzheimer’s Disease, Amyotrophic Lateral Sclerosis (ALS), and peripheral neuropathies. These largely incurable diseases are frequently idiopathic, carry poor prognoses, and impose substantial socioeconomic burdens. Environmental exposures, including repeated pesticide exposures, are also linked to neurodegenerative diseases, but this link is poorly understood [55,57,111,112].

Here, we have evidence of performance limiting health effects from OP pesticide exposure levels below the concentration for the understood mechanism of cholinesterase inhibition. Specifically, we show that repeated exposure to 50 mg MAL/kg body weight is associated with A) no significant changes in blood cholinesterase activity levels, B) sex-specific neurobehavior differences, C) alterations in MN soma sizes, D) evidence of transient peripheral neuropathy, and E) GAL is neuroprotective against MAL effects. Notably, several of these phenotypes parallel features reported in neurodegenerative conditions, raising the possibility that sub-threshold OP exposures engage early or overlapping pathogenic pathways.

Although most of the health effects reported in this study appear transient and recover following cessation of exposure, it is unknown if additional stressors or genetic susceptibility can convert these transient perturbations into persistent dysfunction, or if continued exposure would prolong or exacerbate the observed health effects. These findings underscore the need for longitudinal studies to define the durability and functional consequences of subacute OP exposure, to refine biomonitoring strategies for occupational risk assessment, and to develop mitigation or therapeutic approaches that enable the continued use of pesticides while minimizing short- and long-term neurobiological harm.

## Supporting information

S1 Fig Acetylcholinesterase activity is not altered by malathion exposure. Plasma acetylcholinesterase (AChE) activity did not differ among experimental groups at either the immediate or delayed assessment in males or females. One-way ANOVA detected no significant group effects in (A) males at the immediate time point (*P* = 0.220), (B) males at the delayed time point (*P* = 0.994), (C) females at the immediate time point (*P* = 0.104), or (D) females at the delayed time point (*P* = 0.115). Data are presented as mean ± SEM. Sample sizes: immediate, *n* = 12; delayed, *n* = 6.
(TIF)

S2 Fig Malathion exposure does not induce astrogliosis in the lumbar spinal cord. Astrocytes in the ventral horn of lumbar spinal cord segments L5–L6 were labeled by GFAP immunoreactivity (magenta) in (A) saline controls, (B) malathion (MAL) immediate, and (C) MAL delayed groups. Quantification of GFAP signal revealed no evidence of astrogliosis following MAL exposure. (D, E) GFAP optical density did not differ between treatment groups at either time point in males (immediate: *P* = 0.864; delayed: *P* = 0.937) or females (immediate: *P* = 0.406; delayed: *P* = 0.343). (F, G) Similarly, GFAP pixel intensity showed no group differences at the immediate or delayed assessments in males (immediate: *P* = 0.958; delayed: *P* = 0.949) or females (immediate: *P* = 0.886; delayed: *P* = 0.608). Data are presented as mean ± SEM, with individual animals shown as points. Sample sizes: immediate, *n* = 4; delayed, *n* = 4.
(TIF)

S3 Fig Soma diameter distributions of SK3-positive and SK3-negative motoneurons. Motoneuron (MN) soma diameters were measured for cells exhibiting immunoreactivity (IR) to SK3 and compared with MNs lacking SK3 immunoreactivity. (A–C) Representative images from the lateral lamina IX region of L4–L5 rat spinal cord. MNs were labeled with NeuN (neuronal soma and nuclei), SK3 (membrane channels), and VAChT (C-bouton synapses). Scale bar, 100 µm. (A) Saline control animal. (B) Malathion (MAL)-exposed animal assessed at the immediate time point. (C) MAL-exposed animal assessed at the delayed time point. (D–K) Histograms showing the number of MNs within each soma diameter bin (2-µm intervals; 20–60 µm range). Neurons with diameters <20 µm were excluded from analysis. Distributions are shown separately by sex, time point, and treatment group. Across groups, SK3-negative MNs exhibited a bimodal diameter distribution, whereas SK3-positive MNs showed a unimodal distribution. Statistical comparisons of MN diameters across SK3-reactive and nonreactive populations are provided in Dataset 7.
(TIF)

S4 Fig Fluorescently labeled cross-section of rat lumbar spinal cord. Spinal neurons are stained with fluorescent Nissl (green) and nuclei with DAPI (cyan). Motoneurons are identified as the large diameter neurons within lateral lamina IX (identified by white arrows on each side) in the ventral horns of the grey matter. Motoneurons were quantified in 10 μm confocal z-stacks (z-step = 1.0μm) from confocal microscopy micrographs and were quantified by counting nuclei and measuring soma cross-sectional area through the level of the nucleolus. Scale bar = 1 mm.
(TIF)

S1 File Supplemental Tables S1-S19. (DOCX)
