# Supplementary material for: Performance changes and neuromuscular alterations in sprague dawley rats following repeated occupational exposures to the pesticide malathion
Source: PLoS One. 2026 Sep 15;21(9):e0357900. doi: 10.1371/journal.pone.0357900 (PMC13577405; doi:10.1371/journal.pone.0357900)
Supplement: Supplementary file 1 [file pone.0357900.s005.docx]

**Table of Contents**

Table S1. Male weekly body weights in grams	1

Table S2. Female weekly body weights in grams	2

Table S3. Animal body weight statistics (grams)	4

Table S4. Plasma acetylcholinesterase activity data (nmol/min/ml)	5

Table S5. Plasma acetylcholinesterase (AChE) activity	5

Table S6. Accelerod raw data (seconds)	6

Table S7. Motor activity statistics	6

Table S8. GFAP integrated optical density statistics	8

Table S10. Neuromuscular junction cross-sectional area in extensor digitorum longus and soleus muscles across treatments	8

Table S11. Fragmentation probability – model effects and estimated means	8

Table S12. Motoneuron diameters in galantamine treated groups	11

Table S13. SK3+ Motoneuron diameter sizes in galantamine treated groups	12

Table S14. SK3+ Motoneuron ratio in galantamine treated groups	12

Table S15. Average C-Bouton areas in galantamine treated groups	12

Table S16. Average C-bouton densities in galantamine treated groups	13

Table S17. GFAP integrated optical density in galantamine treated groups	13

Table S18. GFAP mean intensity in galantamine treated groups	14

Table S19. Immunohistochemistry antibodies and approach	14

**Supplementary Data**

# Table S1. Male weekly body weights in grams

|  | **Animal ID** | **2/5** | **2/11** | **2/18** | **2/25** | **3/4** | **3/11** | **3/17** | **3/24** | **4/1** | **4/7** | **4/12** |
| --- | --- | --- | --- | --- | --- | --- | --- | --- | --- | --- | --- | --- |
| **SALINE** | **201** | 108.49 | 186.15 | 240.39 | 284.16 | 318.63 | 351.52 | 373.37 | 385.82 |  |  |  |
|  | **204** | 107.95 | 191.99 | 251.2 | 299.57 | 333.66 | 375.99 | 398.65 | 414.05 |  |  |  |
|  | **212** | 103.82 | 180.15 | 238.33 | 283.76 | 332.65 | 369.57 | 386.76 |  |  |  |  |
|  | **218** | 120.06 | 206.67 | 274.21 | 330.5 | 382.93 | 418.32 | 424.65 |  |  |  |  |
|  | **225** | 123.77 | 207.71 | 269.69 | 316.31 | 349.34 | 382.63 | 403.58 | 418.58 |  |  |  |
|  | **226** | 118.96 | 199.48 | 257.68 | 314.55 | 349.94 | 394.08 | 419.7 |  |  |  |  |
|  | **227** | 106.83 | 187.25 | 245.98 | 293.49 | 334.01 | 363.02 | 386.85 | 413.56 | 436.86 | 446.44 | 469.3 |
|  | **231** | 119.4 | 198.44 | 261.08 | 313.05 | 360.93 | 395.13 | 417.87 | 445.08 | 472.74 | 493.18 | 449.33 |
|  | **233** | 110.71 | 193.48 | 257.47 | 309.11 | 352.75 | 374.12 | 379.9 | 390.04 | 421.84 | 423.59 |  |
|  | **234** | 111.92 | 191.32 | 249.88 | 305.82 | 354.12 | 389.92 | 410.34 | 432.11 | 469.2 | 471.35 |  |
|  | **239** | 109.91 | 204.28 | 271.7 | 328.2 | 377.49 | 420.23 | 448.76 | 485.57 | 521.44 | 534.05 |  |
|  | **246** | 116.37 | 196.27 | 264.52 | 315.52 | 364.41 | 407.14 | 439.9 | 463.39 | 502.53 | 520.12 | 540.89 |
| **MALATHION** | **203** | 112.06 | 196.9 | 259.85 | 312.14 | 362.05 | 393.48 | 415.41 | 435.29 |  |  |  |
|  | **209** | 111.3 | 198.81 | 257.72 | 301.69 | 341.35 | 379.64 | 398.07 | 417.53 |  |  |  |
|  | **216** | 113.81 | 202.42 | 260.74 | 313.33 | 350.62 | 400.57 | 429.38 | 465.64 |  |  |  |
|  | **217** | 113.91 | 193.11 | 253.83 | 302.55 | 335.07 | 373.06 | 385.24 |  |  |  |  |
|  | **219** | 106.79 | 182.46 | 241.25 | 294.88 | 344.73 | 338.1 | 414.65 | 429.9 | 467.87 | 493.64 | 516.57 |
|  | **222** | 125.15 | 209.53 | 270.44 | 315.89 | 359.41 | 405.9 | 421.32 |  |  |  |  |
|  | **224** | 113.86 | 186.24 | 254.9 | 318.56 | 368.1 | 416.96 | 439.6 |  |  |  |  |
|  | **229** | 115.14 | 201.47 | 261.81 | 319.82 | 372.88 | 397.3 | 421.61 | 452.17 | 490.46 | 499.04 |  |
|  | **230** | 121.13 | 211.56 | 284.21 | 340.48 | 394.16 | 444.15 | 460.62 | 515.1 | 563.1 | 576.05 |  |
|  | **235** | 121.97 | 206.85 | 268.52 | 321.29 | 364.89 | 399.82 | 414.56 | 423.89 | 464.99 | 483.46 | 505.41 |
|  | **240** | 105.45 | 189.58 | 250.57 | 293.8 | 326.44 | 364.94 | 399.81 | 420.04 | 447.06 | 460.68 | 479.51 |
|  | **245** | 114.2 | 195.86 | 257.92 | 310.26 | 348.89 | 382.45 | 388.46 | 420.63 | 459.99 | 456.36 |  |
| **GALANTAMINE** | **205** | 122.29 | 213.5 | 271.47 | 327.17 | 374.45 | 416.8 | 435.96 |  |  |  |  |
|  | **206** | 119.06 | 198.67 | 262.51 | 315.65 | 360.55 | 401.83 | 431.52 | 447.71 |  |  |  |
|  | **207** | 132.27 | 214.54 | 272.82 | 315.26 | 347.41 | 378.61 | 406.31 | 436.8 |  |  |  |
|  | **208** | 127.91 | 213.6 | 271.85 | 319.59 | 369.34 | 414.33 | 440.74 |  |  |  |  |
|  | **213** | 113.12 | 198.34 | 250.71 | 303.19 | 351.19 | 382.37 | 396.31 |  |  |  |  |
|  | **221** | 116.05 | 197.13 | 257.55 | 301.13 | 333.29 | 366.3 | 395.5 | 412.12 |  |  |  |
|  | **223** | 111.18 | 186.46 | 241.85 | 297.93 | 339.08 | 367.82 | 348.69 | 416.3 | 438.15 | 439.52 |  |
|  | **238** | 111.96 | 190.33 | 254.17 | 292.35 | 332.52 | 359.58 | 378.99 | 404.42 | 431.21 | 446.05 | 447.72 |
|  | **241** | 123.25 | 212.97 | 264.57 | 327.83 | 380.5 | 433.69 | 462.68 | 515.76 | 555.29 | 572.1 |  |
|  | **242** | 122.65 | 210.33 | 271.66 | 325.18 | 372.5 | 401.15 | 421.99 | 455.06 | 501.99 | 504.5 |  |
|  | **247** | 117.37 | 209.01 | 268.57 | 306.71 | 340.72 | 363.37 | 391.43 | 400.62 | 434.75 | 453.49 | 466.31 |
|  | **248** | 108.32 | 193.76 | 261.55 | 307.13 | 347.35 | 376.48 | 395.38 | 417.48 | 449.29 | 475.44 | 490.8 |
| **MALATHION+GALANTAMINE** | **202** | 111.04 | 191.86 | 249.52 | 303.96 | 350.67 | 379.65 | 396.15 |  |  |  |  |
|  | **210** | 106.73 | 186.64 | 226.91 | 269.07 | 316.36 | 351.5 | 367.35 |  |  |  |  |
|  | **211** | 116.78 | 205.74 | 273.05 | 324.89 | 364.94 | 399.01 | 426.45 | 445.03 |  |  |  |
|  | **214** | 112.39 | 188.64 | 229.86 | 271.96 | 304.85 | 339.17 | 358.13 |  |  |  |  |
|  | **215** | 117.61 | 204.06 | 279.19 | 329.76 | 371.24 | 384.72 | 434.66 | 462.52 |  |  |  |
|  | **220** | 118.06 | 206.85 | 274.04 | 315.67 | 361.33 | 390.4 | 412.6 | 433.37 |  |  |  |
|  | **228** | 122.68 | 204.01 | 256.25 | 308.99 | 339.6 | 371.81 | 392.26 | 438.88 | 475.03 | 485.26 |  |
|  | **232** | 110.27 | 194.22 | 257.06 | 299.18 | 328.88 | 363.24 | 383.85 | 396.1 | 419.99 | 423.74 | 453.62 |
|  | **236** | 119.89 | 207.24 | 242.58 | 281.12 | 318.07 | 349.84 | 361.61 | 395.12 | 434.05 | 451.64 |  |
|  | **237** | 101.63 | 179.63 | 235.44 | 271.35 | 307.1 | 334.78 | 359.92 | 376.26 | 411.95 | 438.39 | 465.27 |
|  | **243** | 119.08 | 207.83 | 272.96 | 319.14 | 370.49 | 407.84 | 430.03 | 450.69 | 493.64 | 507.99 | 520.4 |
|  | **244** | 125.17 | 218.88 | 281.09 | 331.92 | 383.92 | 430.07 | 444.8 | 491.03 | 530.58 | 541.25 |  |

# Table S2. Female weekly body weights in grams

|  | **Animal ID** | **8/16** | **8/26** | **9/2** | **9/9** | **9/16** | **9/23** | **9/29** | **10/6** | **10/14** | **10/20** | **10/27** |
| --- | --- | --- | --- | --- | --- | --- | --- | --- | --- | --- | --- | --- |
| **SALINE** | **305** | 134.49 | 199.24 | 218.86 | 243.55 | 258.04 | 269.13 | 279.3 |  |  |  |  |
|  | **306** | 129.19 | 196.27 | 226.39 | 257.44 | 288.61 | 305.36 | 311.86 | 310.32 |  |  |  |
|  | **309** | 126.11 | 178.98 | 201.89 | 223.36 | 247.23 | 258.25 | 270.5 | 262.85 |  |  |  |
|  | **313** | 126.31 | 192.71 | 220.07 | 252.72 | 288.81 | 331.19 | 315.52 |  |  |  |  |
|  | **320** | 131.48 | 193.31 | 234.7 | 258.57 | 297.91 | 313.59 | 321.84 | 324.38 | 362 | 380.29 | 363.85 |
|  | **324** | 129.64 | 181.83 | 206.61 | 231.48 | 248.05 | 273.75 | 265.83 |  |  |  |  |
|  | **325** | 145.63 | 170.27 | 204.46 | 233.37 | 253.15 | 256.8 | 258.82 | 262.36 | 280.34 | 275.51 |  |
|  | **336** | 126 | 178.41 | 208.79 | 230.46 | 252.24 | 266.06 | 277.82 | 272.02 |  |  |  |
|  | **338** | 122.68 | 186.01 | 220.37 | 248.53 | 277.09 | 293.45 | 294.16 | 311.46 | 333.89 | 331.6 |  |
|  | **340** | 117.63 | 188.36 | 210.03 | 212.17 | 251.7 | 275.44 | 274.4 | 290.05 | 305.17 | 307.57 |  |
|  | **341** | 126.59 | 195.61 | 226.64 | 266.7 | 290.19 | 319.96 | 331.03 | 335.76 | 369.11 | 376.68 | 385.06 |
|  | **343** | 128.47 | 180.26 | 97.29 | 217.21 | 237.32 | 258.78 | 269.28 | 263.2 | 277.37 | 285.51 | 288.93 |
| **MALATHION** | **302** | 131.89 | 191.38 | 231.3 | 256.55 | 266.32 | 290.63 | 288.11 | 301.74 |  |  |  |
|  | **304** | 145.62 | 202.83 | 256.35 | 249.46 | 267.37 | 279.78 | 294.21 | 292.71 |  |  |  |
|  | **310** | 125.27 | 176.69 | 206.57 | 220.01 | 237.66 | 252.14 | 254.92 | 255.58 | 345.67 | 345.67 | 343.07 |
|  | **314** | 113.66 | 183.08 | 204.55 | 220.2 | 234.73 | 250.59 | 256.81 |  |  |  |  |
|  | **315** | 128.12 | 185.93 | 214.85 | 237.38 | 257.69 | 289.09 | 299.76 |  |  |  |  |
|  | **318** | 138.46 | 201.71 | 225.58 | 248.46 | 270.86 | 300.15 | 317.35 | 311.76 | 340.87 | 346 |  |
|  | **321** | 136.87 | 200.87 | 240.83 | 259.24 | 274.77 | 282.53 | 295.77 | 294.65 | 312.92 | 323.54 | 320.24 |
|  | **328** | 119.85 | 172.2 | 193.52 | 211.89 | 230.09 | 250.19 | 251.92 |  |  |  |  |
|  | **334** | 117.85 | 192.17 | 224.98 | 246.88 | 276.28 | 286.12 | 294.69 | 290.83 | 304.95 | 313.64 | 312.84 |
|  | **342** | 128.69 | 179.98 | 207.34 | 227.69 | 246 | 268.42 | 261.62 | 272.04 | 285.38 | 280.99 |  |
|  | **344** | 121.55 | 178.9 | 203.14 | 222.34 | 242.03 | 257.92 | 261.46 | 282.22 | 302.14 | 303.97 |  |
|  | **347** | 117.49 | 171.1 | 194.52 | 209.47 | 229.57 | 247.21 | 244.25 | 266.56 | 281.16 | 287.87 |  |
| **GALANTAMINE** | **301** | 129.69 | 193.88 | 217.4 | 246.86 | 260.58 | 284.72 | 286.44 |  |  |  |  |
|  | **307** | 118.85 | 176.08 | 196.08 | 219.31 | 240.26 | 259.64 | 253.06 |  |  |  |  |
|  | **312** | 126.37 | 200.65 | 230.34 | 256.77 | 281.38 | 293.19 | 305.12 | 300.87 |  |  |  |
|  | **319** | 135.27 | 184.78 | 205.21 | 223.37 | 247.96 | 258.98 | 273.12 | 278.73 |  |  |  |
|  | **323** | 130.14 | 195.53 | 228.4 | 250.51 | 296.24 | 307.14 | 305.21 |  |  |  |  |
|  | **326** | 133.4 | 154.9 | 200.01 | 230.04 | 251.81 | 263.43 | 264.58 |  |  |  |  |
|  | **327** | 131.72 | 201.97 | 232.66 | 255.89 | 276.78 | 292.69 | 300.68 | 302.06 | 315.17 | 327.65 | 323.27 |
|  | **330** | 134.41 | 186.5 | 217.62 | 235.94 | 255.74 | 272.4 | 272.94 | 287.5 | 288.22 | 290.41 | 289.62 |
|  | **337** | 142.85 | 196.96 | 215.87 | 243.85 | 266.59 | 281.3 | 293.82 | 306.41 | 335.57 | 341.92 |  |
|  | **339** | 129.84 | 187.83 | 217.36 | 234.2 | 256.89 | 269.44 | 272.02 | 279.03 | 282.03 | 284.51 | 307 |
|  | **346** | 137.11 | 199.33 | 223.74 | 238.36 | 256.34 | 273.62 | 270.05 | 290.3 | 314.14 | 313.14 |  |
|  | **348** | 126.31 | 181.83 | 200.68 | 220.82 | 236.12 | 251.56 | 250.86 | 269.4 | 290.7 | 281.94 |  |
| **MALATHION+GALANTAMINE** | **303** | 136.05 | 177.89 | 198.63 | 209.85 | 228.85 | 240.33 | 240.49 |  |  |  |  |
|  | **308** | 128.19 | 189.32 | 223.29 | 246.72 | 273.07 | 283.26 | 299.9 |  |  |  |  |
|  | **311** | 136.4 | 197.03 | 211.47 | 227.69 | 235.76 | 257.99 | 261.27 | 269.61 |  |  |  |
|  | **316** | 126.91 | 189.56 | 223.1 | 244.87 | 254.7 | 270.71 | 280.78 | 278.74 |  |  |  |
|  | **317** | 119.35 | 186.56 | 207.3 | 229.54 | 245.87 | 266.89 | 278.52 |  |  |  |  |
|  | **322** | 127.65 | 201.41 | 224.64 | 252.11 | 272.69 | 283.7 | 297.99 | 291.5 | 331.43 | 343.43 |  |
|  | **329** | 123.76 | 192.18 | 218.96 | 238.7 | 255.12 | 265.57 | 273.27 | 271.6 |  |  |  |
|  | **331** | 136.08 | 195.98 | 228.93 | 268.49 | 290.11 | 319.39 | 317.56 | 344.61 | 390.13 | 383.01 |  |
|  | **332** | 131.29 | 179 | 207.98 | 223.57 | 245.19 | 259.2 | 272.92 | 273.45 | 292.39 | 300.85 | 302.24 |
|  | **333** | 124.95 | 182.02 | 206.44 | 228.51 | 240.86 | 250.26 | 254.16 | 267.3 | 287.77 | 289.57 |  |
|  | **335** | 128.68 | 186.59 | 217.03 | 246.45 | 267.46 | 276.12 | 219.14 | 295.17 | 318.49 | 315.06 | 319.12 |
|  | **345** | 137.15 | 193.63 | 224.91 | 241.62 | 257.28 | 273.49 | 283.89 | 261 | 319.55 | 318.87 | 330.35 |

# Table S3. Animal body weight statistics (grams)

|  | **Date** | **Saline** | | | **MAL** | | | **GAL** | | | **MAL + GAL** | | | **ANOVA** |
| --- | --- | --- | --- | --- | --- | --- | --- | --- | --- | --- | --- | --- | --- | --- |
|  |  | *Mean* ± *SEM* | | | *Mean* ± *SEM* | | | *Mean* ± *SEM* | | | *Mean* ± *SEM* | | | **P values** |
| **Males** | **2/5/2022** | 113.18 | ± | 1.82 | 114.56 | ± | 1.68 | 118.79 | ± | 2.07 | 115.11 | ± | 1.97 | 0.20 |
|  | **2/11/2022** | 195.27 | ± | 2.46 | 197.90 | ± | 2.62 | 203.22 | ± | 2.94 | 199.63 | ± | 3.26 | 0.26 |
|  | **2/18/2022** | 256.84 | ± | 3.46 | 260.15 | ± | 3.12 | 262.44 | ± | 2.83 | 256.50 | ± | 5.67 | 0.74 |
|  | **2/25/2022** | 307.84 | ± | 4.40 | 312.06 | ± | 3.74 | 311.59 | ± | 3.44 | 302.25 | ± | 6.79 | 0.82 |
|  | **3/4/2022** | 350.91 | ± | 5.50 | 355.72 | ± | 5.33 | 354.08 | ± | 4.87 | 343.12 | ± | 7.97 | 0.47 |
|  | **3/11/2022** | 386.81 | ± | 6.19 | 391.36 | ± | 7.75 | 388.53 | ± | 7.03 | 375.17 | ± | 8.38 | 0.44 |
|  | **3/17/2022** | 407.53 | ± | 6.85 | 415.73 | ± | 6.20 | 408.79 | ± | 8.99 | 397.32 | ± | 9.18 | 0.44 |
|  | **3/24/2022** | 427.58 | ± | 10.92 | 442.24 | ± | 10.58 | 434.03 | ± | 12.00 | 432.11 | ± | 12.21 | 0.84 |
|  | **4/1/2022** | 470.77 | ± | 15.41 | 482.25 | ± | 17.17 | 468.45 | ± | 20.39 | 460.87 | ± | 19.08 | 0.81 |
|  | **4/7/2022** | 481.46 | ± | 17.39 | 494.87 | ± | 17.69 | 481.85 | ± | 20.47 | 474.71 | ± | 18.34 | 0.89 |
|  | **4/12/2022** | 486.51 | ± | 27.80 | 500.50 | ± | 10.98 | 468.28 | ± | 12.47 | 479.76 | ± | 20.59 | 0.70 |
| **Females** | **8/16/2022** | 128.69 | ± | 1.97 | 127.11 | ± | 2.81 | 131.33 | ± | 1.76 | 129.71 | ± | 1.66 | 0.55 |
|  | **8/26/2022** | 186.77 | ± | 2.57 | 186.40 | ± | 3.27 | 188.35 | ± | 3.84 | 189.26 | ± | 2.10 | 0.90 |
|  | **9/2/2022** | 206.34 | ± | 10.33 | 216.96 | ± | 5.55 | 215.45 | ± | 3.59 | 216.06 | ± | 2.74 | 1.00 |
|  | **9/9/2022** | 239.63 | ± | 5.06 | 234.13 | ± | 5.09 | 237.99 | ± | 3.78 | 238.18 | ± | 4.44 | 0.86 |
|  | **9/16/2022** | 265.86 | ± | 6.08 | 252.78 | ± | 5.19 | 260.56 | ± | 5.00 | 255.58 | ± | 5.12 | 0.42 |
|  | **9/23/2022** | 285.15 | ± | 7.63 | 271.23 | ± | 5.47 | 275.68 | ± | 4.78 | 270.58 | ± | 5.78 | 0.30 |
|  | **9/29/2022** | 289.20 | ± | 7.13 | 276.74 | ± | 6.91 | 278.99 | ± | 5.50 | 273.32 | ± | 7.78 | 0.41 |
|  | **10/6/2022** | 292.49 | ± | 9.60 | 285.34 | ± | 5.97 | 289.29 | ± | 4.64 | 283.66 | ± | 8.47 | 0.84 |
|  | **10/14/2022** | 321.31 | ± | 16.30 | 310.44 | ± | 9.46 | 304.31 | ± | 8.43 | 323.29 | ± | 15.04 | 0.69 |
|  | **10/20/2022** | 326.19 | ± | 18.33 | 314.53 | ± | 9.75 | 306.60 | ± | 10.15 | 325.13 | ± | 13.76 | 0.70 |
|  | **10/27/2022** | 345.95 | ± | 29.16 | 325.38 | ± | 9.10 | 306.63 | ± | 9.72 | 317.24 | ± | 8.17 | 0.44 |

# Table S4. Plasma acetylcholinesterase activity data (nmol/min/ml)

|  | **MALES** | | | | | | | | | |
| --- | --- | --- | --- | --- | --- | --- | --- | --- | --- | --- |
|  | **Immediate Timepoint** | | | | | | **Delayed Timepoint** | | | |
|  | **Saline** | | **Malathion** | | **Galantamine** | **Malathion + Galantamine** | **Saline** | **Malathion** | **Galantamine** | **Malathion + Galantamine** |
| 7.03 | | 4.31 | | 5.43 | | 5.99 | 5.66 | 5.30 | 6.88 | 3.70 |
| 3.12 | | 6.20 | | 4.82 | | 4.87 | 5.89 | 2.56 | 3.49 | 5.11 |
| 4.24 | | 5.05 | | 5.72 | | 1.71 | 4.78 | 1.80 | 4.16 | 2.40 |
| 2.64 | | 5.41 | | 1.22 | | 2.19 | 1.85 | 5.77 | 3.37 | 7.89 |
| 6.08 | | 6.29 | | 5.45 | | 3.38 | 3.66 | 6.98 | 7.89 | 3 |
| 8.44 | | 7.27 | | 1.96 | |  |  | 5.69 | 1.43 |  |
|  | **FEMALES** | | | | | | | | | |
| 3.69 | | 10.06 | | 5.19 | | 9.06 | 8.06 | 10.52 | 9.42 | 6.04 |
| 9.14 | | 8.45 | | 8.81 | | 6.30 | 10.35 | 8.34 | 6.99 | 5.24 |
| 6.07 | | 9.42 | | 12.16 | | 7.20 | 8.71 | 9.14 | 6.96 | 5.66 |
| 6.23 | | 5.56 | | 11.47 | | 4.54 | 9.13 | 6.48 | 10.66 | 4.77 |
| 5.19 | | 10.65 | | 12.94 | | 5.97 | 6.02 | 7.83 | 6.13 | 10.17 |
| 5.56 | | 5.34 | | 5.40 | | 7.93 | 7.80 | 10.52 | 9.54 | 6.76 |

# Table S5. Plasma acetylcholinesterase (AChE) activity

| **Time Point** | **Group** | **n** | **AChE Activity (nmol·min⁻¹·mL⁻¹),** |
| --- | --- | --- | --- |
| Immediate | Saline Male | 6 | 5.26 ± 2.30 |
| Immediate | MAL Male | 6 | 5.75 ± 1.05 |
| Immediate | Saline Female | 5 | 5.98 ± 1.79 |
| Immediate | MAL Female | 6 | 8.25 ± 2.29 |
| Delayed | Saline Male | 5 | 4.37 ± 1.66 |
| Delayed | MAL Male | 6 | 4.68 ± 2.03 |
| Delayed | Saline Female | 6 | 8.35 ± 1.45 |
| Delayed | MAL Female | 6 | 8.81 ± 1.58 |

Data is presented as mean ± standard deviation with n = animal number. Two-tailed Student’s *t* tests were used to compare saline and malathion (MAL) groups within each sex and time point. Immediate: males *P* = 0.643, females *P* = 0.085. Delayed: males *P* = 0.785, females *P* = 0.611.

# Table S6. Accelerod raw data (seconds)

| **MALES** | | | | | | | |
| --- | --- | --- | --- | --- | --- | --- | --- |
| **Immediate Timepoint** | | | | **Delayed Timepoint** | | | |
| **Saline** | **Malathion** | **Galantamine** | **Malathion + Galantamine** | **Saline** | **Malathion** | **Galantamine** | **Malathion + Galantamine** |
| 179.9 | 159.9 | 137.9 | 207.2 | 133.22 | 204.24 | 159.84 | 182.47 |
| 204.9 | 78.6 | 145.6 | 163.8 | 174.5 | 152.61 | 217.11 | 193.83 |
| 178.1 | 112.2 | 158.7 | 153 | 106.02 | 130.94 | 172.77 | 165.08 |
| 147.4 | 136.3 | 186.8 | 157.7 | 183.94 | 142.7 | 180.67 | 158.17 |
| 226.2 | 156.7 | 223.4 | 199.8 | 114.7 | 133.03 | 139.52 | 83.61 |
| 152.9 | 82.3 | 151.6 | 166.8 | 93.02 | 85.78 | 191.64 | 175.45 |
| 205 | 116 | 114 | 169 |  |  |  |  |
| 217.8 | 75.6 | 152.3 | 109.1 |  |  |  |  |
| 139.3 | 119.9 | 163.3 | 91.9 |  |  |  |  |
| 184.1 | 186.6 | 156.9 | 154.1 |  |  |  |  |
| 191 | 197.4 | 115.5 | 163.4 |  |  |  |  |
| 173.2 | 122.6 | 189.2 | 232.2 |  |  |  |  |
| **FEMALES** | | | | | | | |
| 218.89 | 251.38 | 262.47 | 289.55 | 252.34 | 300 | 229.2 | 151.06 |
| 251.97 | 284.22 | 267.36 | 229.33 | 186.5 | 234.38 | 212.84 | 204.83 |
| 227 | 231.09 | 173.17 | 276.69 | 126.99 | 192.09 | 141.88 | 228.25 |
| 216.52 | 213.7 | 229.83 | 265.06 | 280.94 | 79.27 | 218.39 | 275.56 |
| 176.61 | 177.94 | 245.75 | 163.69 | 170.91 | 213.28 | 43.41 | 268.41 |
| 256.39 | 233.13 | 173.38 | 258.45 | 163.39 | 202.03 | 142.19 | 275.47 |
| 272.25 | 219.24 | 177.44 | 278.88 |  |  |  |  |
| 295.81 | 174.02 | 246.11 | 145.22 |  |  |  |  |
| 168.55 | 300 | 222.17 | 289.94 |  |  |  |  |
| 222.03 | 188.78 | 148.84 | 244.38 |  |  |  |  |
| 206.61 | 148.58 | 76.24 | 157.77 |  |  |  |  |
| 236.5 | 271.36 | 285.41 | 300 |  |  |  |  |

# Table S7. Motor activity statistics

| **Male Immediate Motor Activity Parameters** | | | | | |
| --- | --- | --- | --- | --- | --- |
| **Measurement** | **Saline** | **Malathion** | **Galantamine** | **Malathion + Galantamine** | **ANOVA p-values** |
| **Ambulatory Beam Breaks (occurrences)** | 7081.92 ± 1063.52, 307.01 | 5203.67 ± 974.38, 281.28 | 6185.42 ± 1296.35, 374.22 | 6946.5 ± 1055.58, 304.72 | Saline vs. MAL p <0.001  MAL vs. MAL+GAL p = 0.002 |
| **Stereotypical Beam Breaks**  **(occurrences)** | 231.92 ± 14.07, 4.06 | 228.5 ± 14.85, 4.29 | 218 ± 21.71, 6.27 | 219.5 ± 12.77, 3.69 | p = 0.114 |
| **% Time in Center** | 46.4 ± 4.37, 1.26 | 46.70 ± 2.83, 0.82 | 46.71 ± 5.09, 1.47 | 46.37 ± 3.93, 1.14 | p = 0.995 |
| **Total Rears**  **(occurrences)** | 195.5 ± 41.23, 11.90 | 149.83 ± 46.64, 13.46 | 158.08 ± 45.62, 13.17 | 173 ± 26.91, 7.77 | Saline vs. MAL p = 0.046 |
| **Speed (cm/sec)** | 36.99 ± 2.28, 0.66 | 36.22 ± 2.64, 0.76 | 36.11 ± 3.02, 0.87 | 37.91 ± 2.44, 0.70 | p = 0.31 |
| **Active time (sec)** | 1488.25 ± 150.38, 43.41 | 1253.16 ± 151.06, 43.61 | 1353.42 ± 204.44, 59.02 | 1461.21 ± 109.72, 31.67 | Saline vs. MAL p = 0.004  MAL vs. MAL+GAL p = 0.012 |
| **Total Distance (cm)** | 39183.68 ± 5845.86, 1687.55 | 30541.64 ± 5666.16, 1635.68 | 34969.63 ± 7485.88, 2160.99 | 39160.65 ± 5727.27, 1653.32 | Saline vs. MAL p = 0.009  MAL vs. MAL+GAL p = 0.007 |
| **Male Delayed Motor Activity Parameters** | | | | | |
| **Ambulatory Beam Breaks**  **(occurrences)** | 6700.67 ± 1478.03, 603.40 | 5555.83 ± 762.80, 311.41 | 6354.83 ± 1192.54, 486.85 | 7489.5 ± 803.38, 327.98 | MAL+GAL vs. MAL p = 0.038 |
| **Stereotypical Beam Breaks**  **(occurrences)** | 207.33 ± 29.14, 11.90 | 225.17 ± 11.62, 4.74 | 229.17 ± 15.96, 6.51 | 220 ± 15.28, 6.24 | p = 0.253 |
| **% Time in Center** | 50.45 ± 7.92, 3.23 | 51.80 ± 3.67, 1.50 | 49.94 ± 4.30, 1.76 | 52.62 ± 3.30, 1.35 | p = 0.794 |
| **Total Rears**  **(occurrences)** | 167.17 ± 26.74, 10.92 | 134.67 ± 46.57, 19.01 | 132.5 ± 36.48, 14.89 | 174 ± 48.14, 19.65 | p = 0.197 |
| **Speed (cm/sec)** | 39.10 ± 1.59, 0.65 | 40.45 ± 2.36, 0.96 | 37.57 ± 2.78, 1.14 | 42.41 ± 2.12, 0.86 | GAL vs. MAL+GAL p = 0.008 |
| **Active time (sec)** | 1446.4 ± 186.50, 76.14 | 1320.2 ± 97.13, 39.65 | 1407.8 ± 192.74, 78.69 | 1520.52 ± 100.92, 41.20 | p = 0.176 |
| **Total Distance (cm)** | 39306.86 ± 8450.63, 3450.00 | 34305.94 ± 4863.70, 1985.60 | 36518.22 ± 6720.39, 2743.59 | 45208.10 ± 5686.18, 2321.38 | p = 0.05 |
| **Female Immediate Motor Activity Parameters** | | | | | |
| **Ambulatory Beam Breaks**  **(occurrences)** | 5457.25 ± 886.29, 255.85 | 4321.17 ± 945.03, 272.81 | 5623.92 ± 1096.5, 316.53 | 5149.67 ± 1117.17, 322.5 | Saline vs. MAL p = 0.044  GAL vs. MAL p =0.018 |
| **Stereotypical Beam Breaks**  **(occurrences)** | 264.5 ± 27.18, 7.85 | 223.08 ± 51.74, 14.94 | 254.25 ± 23.34, 6.74 | 244.5 ± 33.97, 9.81 | Saline vs. MAL p = 0.04 |
| **% Time in Center** | 34.61 ± 3.1, 0.90 | 37.95 ± 4.04, 1.17 | 38.03 ± 7.1, 2.05 | 37.49 ± 4.24, 1.23 | p = 0.274 |
| **Total Rears**  **(occurrences)** | 182.33 ± 42.50, 12.27 | 140.33 ± 47.83, 13.81 | 191.08 ± 49.76, 14.36 | 172.67 ± 49.22, 14.21 | p = 0.062 |
| **Speed (cm/sec)** | 32.56 ± 1.75, 0.50 | 30.37 ± 5.34, 1.54 | 32.94 ± 2.87, 0.83 | 32.46 ± 2.46, 0.71 | p = 0.272 |
| **Active time (sec)** | 1356.68 ± 122.15, 35.26 | 1101.47 ± 218.27, 63.01 | 1353.86 ± 150.48, 43.44 | 1265.15 ± 197.63, 57.05 | Saline vs. MAL p = 0.006  GAL vs. MAL p = 0.005 |
| **Total Distance (cm)** | 28059.79 ± 4273.13, 1233.55 | 22519.28 ± 5066.82, 1462.67 | 28866.69 ± 5546.97, 1601.27 | 26569.37 ± 6321.62, 1824.89 | GAL vs. MAL p = 0.034 |
| **Female Delayed Motor Activity Parameters** | | | | | |
| **Ambulatory Beam Breaks**  **(occurrences)** | 4573.17 ± 956.91, 390.66 | 4439.67 ± 636.80, 259.96 | 5613 ± 1236.64, 504.85 | 5309.67 ± 1365.27, 557.37 | p = 0.212 |
| **Stereotypical Beam Breaks**  **(occurrences)** | 247.83 ± 14.97, 6.11 | 216.5 ± 15.83, 6.46 | 234.17 ± 36.05, 14.72 | 233 ± 21.22, 8.66 | p = 0.184 |
| **% Time in Center** | 41.07 ± 8.37, 3.42 | 41.08 ± 7.63, 3.12 | 39.03 ± 6.01, 2.45 | 42.49 ± 5.23, 2.13 | p = 0.857 |
| **Total Rears**  **(occurrences)** | 115.33 ± 41.53, 16.96 | 112.67 ± 34.00, 13.88 | 152.67 ± 61.58, 25.14 | 158.33 ± 47.84, 19.53 | p = 0.231 |
| **Speed (cm/sec)** | 33.88 ± 3.80, 1.55 | 34.02 ± 1.97, 0.80 | 35.33 ± 2.17, 0.88 | 33.86 ± 2.11, 0.86 | p = 0.728 |
| **Active time (sec)** | 1180.37 ± 186.31, 76.06 | 1148.88 ± 150.41, 61.40 | 1291.23 ± 228.46, 93.27 | 1262.18 ± 218.90, 89.37 | p = 0.572 |
| **Total Distance (cm)** | 25789.34 ± 5689.40, 2322.69 | 24600.72 ± 3699.53, 1510.33 | 30021.11 ± 5665.42, 2312.90 | 27965.39 ± 6841.68, 2793.10 | p = 0.371 |

# Table S8. GFAP integrated optical density statistics

| **Immediate** | | | | | | | |
| --- | --- | --- | --- | --- | --- | --- | --- |
|  | **Saline Male** | **MAL Male** | **Saline Female** | **MAL Female** |  | **Student’s t-test** | |
|  | n = 6 | n = 6 | n = 4 | n = 4 |  | Male:  Saline vs MAL | Female:  Saline vs MAL |
|  | Mean ± SD, SEM | | | |  |  |  |

# Table S10. Neuromuscular junction cross-sectional area in extensor digitorum longus and soleus muscles across treatments

| **Sex** | **Muscle** | **Saline**  **(µm^2^)** | **iMAL**  **(µm^2^)** | **dMAL**  **(µm^2^)** | **P-Value** | **n (Saline/ iMAL/ dMAL)** |
| --- | --- | --- | --- | --- | --- | --- |
| Male | EDL | 809.4 ± 116.2 | 814.8 ± 129.5 | 945.7 ± 144.2 | 0.266 | 120 / 118 / 120 |
| Male | Soleus | 1055.8 ± 124.2 | 934.6 ± 100.0 | 949.2 ± 341.6 | 0.106 | 140 / 120 / 120 |
| Female | EDL | 736.9 ± 109.9 | 726.2 ± 108.0 | 851.3 ± 141.5 | 0.117 | 120 / 100 / 120 |
| Female | Soleus | 983.0 ± 243.7 | 912.4 ±  94.6 | 974.5 ± 150.7 | 0.691 | 119 / 100 / 120 |
| Values are mean ± SD. NMJ area (µm²) was calculated either as the average per animal or per individual NMJ as indicated. n = the number of NMJs analyzed from 6 animals from each group with the exception of 7 animals for the male soleus saline group, and 5 for the female EDL iMAL and female soleus iMAL groups. Muscles examined include predominately fast type the extensor digitorum longus (EDL) and the predominately slow type soleus. P values were calculated using generalized estimating equation models with exposure group as a fixed effect and animal ID as the clustering variable to account for repeated NMJ measurements within animals. | | | | | | |

# Table S11. Fragmentation probability – model effects and estimated means

| **Binomial Logistic Mixed-Effects Model Terms** | | | | | | | |
| --- | --- | --- | --- | --- | --- | --- | --- |
| **Biological Effect** | **Change in Fragmentation Log-Odds ± SE** | **p-value** | **Effect Meaning** | | | | |
| **Baseline - Female Effects** | | | | | | | |
| NMJ Area  (per +1 SD) | 2.19 ± 0.68 | p = 0.001 | In Saline females (EDL), larger NMJs strongly increase fragmentation odds. | | | | |
| dMAL vs Saline  (EDL females) | 2.34 ± 0.68 | p < 0.001 | In EDL females at mean area, dMAL significantly increases fragmentation vs Saline. | | | | |
| iMAL vs Saline (EDL females) | 2.25 ± 0.71 | p = 0.001 | In EDL females at mean area, iMAL significantly increases fragmentation vs Saline. | | | | |
| Soleus × dMAL | -2.46 ± 0.71 | p < 0.001 | The dMAL-associated increase in fragmentation seen in EDL females is markedly reduced in Soleus. | | | | |
| Soleus × iMAL | -2.51 ± 0.73 | p < 0.001 | The iMAL-associated increase in fragmentation in EDL females is markedly reduced in Soleus. | | | | |
| NMJ Area × dMAL | -1.21 ± 0.73 | p = 0.098 | In EDL females, dMAL tends to weaken the size-fragmentation relationship (trend only). | | | | |
| **Male Modifiers of Treatment and Size Effects** | | | | | | | |
| NMJ Area × Male | -2.13 ± 0.75 | p = 0.005 | In Saline EDL, males show a significantly weaker size-dependent increase in fragmentation than females. | | | | |
| dMAL × Male | -1.70 ± 0.81 | p = 0.036 | The dMAL-induced fragmentation increase in EDL is smaller in males than females. | | | | |
| iMAL × Male | -2.71 ± 0.86 | p = 0.002 | The iMAL-induced fragmentation increase in EDL is substantially smaller in males vs females. | | | | |
| NMJ Area × dMAL × Male | 2.05 ± 0.84 | p = 0.014 | In EDL males, dMAL significantly modifies the size-dependent fragmentation slope. | | | | |
| **Model fitted Estimates and Slopes** | | | | | | | |
| **Male - Fragmentation Probability across NMJ area** | | | | | | | |
| **Global NMJ Area** | **EDL** | | | | | | |
|  | **SAL** | **iMAL** | **dMAL** |  | **Wald z-test** | | |
|  | n = 120 | n = 120 | n = 120 |  |  |  |  |
|  | Mean [95% CI] | | |  | SAL v dMAL | SAL v iMAL | iMAL v dMAL |
| -1 SD | 0.127  [0.06, 0.249] | 0.054  [0.02, 0.136] | 0.106  [0.047, 0.223] |  | p = 0.943 | p = 0.339 | p = 0.529 |
| Mean | 0.133  [0.075, 0.223] | 0.088  [0.044, 0.166] | 0.225  [0.139, 0.344] |  | p = 0.308 | p = 0.610 | p = 0.052 |
| +1 SD | 0.139  [0.059, 0.293] | 0.14  [0.061, 0.289] | 0.416  [0.276, 0.571] |  | **p = 0.027** | p = 1.000 | **p = 0.024** |
| Slope (Fold-change/SD) | 1.054  [0.562, 1.977] | 1.694  [0.895, 3.205] | 2.451  [1.501, 4.004] |  | p = 0.095 | p = 0.553 | p = 0.639 |
| **Global NMJ Area** | **Soleus** | | | | | | |
|  | **SAL** | **iMAL** | **dMAL** |  | **Wald z-test** | | |
|  | n = 140 | n = 120 | n = 120 |  |  |  |  |
|  | Mean [95% CI] | | |  | SAL v dMAL | SAL v iMAL | iMAL v dMAL |
| -1 SD | 0.195  [0.107, 0.329] | 0.106  [0.046, 0.224] | 0.129  [0.067, 0.233] |  | p = 0.595 | p = 0.430 | p = 0.923 |
| Mean | 0.299  [0.205, 0.413] | 0.248  [0.158, 0.366] | 0.222  [0.14, 0.333] |  | p = 0.550 | p = 0.781 | p = 0.934 |
| +1 SD | 0.428  [0.308, 0.558] | 0.478  [0.305, 0.657] | 0.355  [0.219, 0.52] |  | p = 0.762 | p = 0.899 | p = 0.580 |
| Slope (Fold-change/SD) | 1.758  [1.225, 2.523] | 2.787  [1.539, 5.047] | 1.933  [1.279, 2.921] |  | p = 0.938 | p = 0.397 | p = 0.586 |
| **Female - Fragmentation Probability across NMJ area** | | | | | | | |
|  | **EDL** | | | | | | |
| **Global NMJ Area** | **SAL** | **iMAL** | **dMAL** |  | **Wald z-test** | | |
|  | n = 120 | n = 120 | n = 120 |  |  |  |  |
|  | Mean [95% CI] | | |  | SAL v dMAL | SAL v iMAL | iMAL v dMAL |
| -1 SD | 0.004  [0, 0.039] | 0.064  [0.024, 0.159] | 0.127  [0.062, 0.244] |  | **p = 0.011** | p = 0.071 | p = 0.487 |
| Mean | 0.036  [0.011, 0.111] | 0.261  [0.154, 0.407] | 0.278  [0.183, 0.399] |  | **p = 0.002** | **p = 0.004** | p = 0.978 |
| +1 SD | 0.249  [0.094, 0.514] | 0.646  [0.371, 0.85] | 0.506  [0.333, 0.677] |  | p = 0.237 | p = 0.098 | p = 0.674 |
| Slope (Fold-change/SD) | 8.898  [2.34, 33.838] | 5.165  [2.219, 12.02] | 2.654  [1.567, 4.494] |  | p = 0.223 | p = 0.778 | p = 0.389 |
|  | **Soleus** | | | | | | |
| **Global NMJ Area** | **SAL** | **iMAL** | **dMAL** |  | **Wald z-test** | | |
|  | n = 120 | n = 100 | n = 120 |  |  |  |  |
|  | Mean [95% CI] | | |  | SAL v dMAL | SAL v iMAL | iMAL v dMAL |
| -1 SD | 0.157  [0.075, 0.299] | 0.215  [0.109, 0.379] | 0.166  [0.081, 0.309] |  | p = 0.993 | p = 0.787 | p = 0.846 |
| Mean | 0.426  [0.3, 0.562] | 0.365  [0.246, 0.503] | 0.395  [0.276, 0.529] |  | p = 0.944 | p = 0.797 | p = 0.942 |
| +1 SD | 0.748  [0.578, 0.865] | 0.546  [0.371, 0.71] | 0.683  [0.524, 0.808] |  | p = 0.811 | p = 0.210 | p = 0.471 |
| Slope (Fold-change/SD) | 3.996  [2.227, 7.172] | 2.094  [1.264, 3.468] | 3.291  [1.971, 5.497] |  | p = 0.229 | p = 0.434 | p = 0.095 |

All values shown are derived from the binomial logistic mixed-effects model. The log-odds can be interpreted as either an increase (positive) or decrease (negative) in the probability of NMJ fragmentation, compared to the reference group (female, EDL, saline, mean NMJ area). Only the significant terms from the model are shown for brevity.

# Table S12. Motoneuron diameters in galantamine treated groups

| **Mean of entire Population - Male Immediate** | | | | | | |
| --- | --- | --- | --- | --- | --- | --- |
|  | **Saline** | **MAL** | **GAL** | **MAL + GAL** |  | **Kruskal Wallis ANOVA; Dunn’s Method** |
|  | n = 538 | n = 615 | n = 357 | n = 407 |  |  |
|  | Mean ± SD, SEM | | | |  |  |
| **Diameters (μm)** | 39.01 ± 9.72, 0.42 | 38.67 ± 10.73, 0.43 | 39.15 ± 8.46, 0.45 | 41.16 ± 9.33, 0.46 |  | MAL+GAL vs. Saline p = 0.005  MAL+GAL vs. GAL p = 0.001  MAL+GAL vs. MAL p = 0.004 |
| **Mean of entire Population - Male Delayed** | | | | | | |
|  | **Saline** | **MAL** | **GAL** | **MAL + GAL** |  | **Kruskal Wallis ANOVA; Dunn’s Method** |
|  | n = 419 | n = 615 | n = 333 | n = 372 |  |  |
|  | Mean ± SD, SEM | | | |  |  |
| **Diameters (μm)** | 39.59 ± 10.22, 0.50 | 40.46 ± 10.22, 0.46 | 39.78 ± 9.79, 0.54 | 42.42 ± 8.63, 0.45 |  | MAL+GAL vs. Saline p = 0.001  MAL+GAL vs. GAL p = 0.003  MAL+GAL vs. MAL p = 0.058 |
| **Means of size subpopulations - Male Immediate** | | | | | | |
|  | **Saline** | **MAL** | **GAL** | **MAL + GAL** |  | **Kruskal Wallis ANOVA; Dunn’s Method** |
|  | Mean ± SD, SEM | | | |  |  |
| **Small-sized Diameters** **(μm)** | 24.74 ± 3.05, 0.25 | 25.29 ± 3.72, 0.26 | 25.04 ± 3.03, 0.35 | 25.23 ± 3.41, 0.38 |  | p = 0.706 |
| **n** | 144 | 207 | 73 | 79 |  |  |
| **Large-sized Diameters** **(μm)** | 44.23 ± 4.87, 0.25 | 45.46 ± 5.42, 0.27 | 42.78 ± 4.82, 0.29 | 45.00 ± 5.40, 0.30 |  | Saline vs. MAL p = 0.004  Saline vs. GAL p < 0.001  GAL vs. MAL p < 0.001  GAL vs. MAL+GAL p < 0.001 |
| **n** | 394 | 408 | 284 | 328 |  |  |
| **Means of size subpopulations - Male Delayed** | | | | | | |
|  | **Saline** | **MAL** | **GAL** | **MAL + GAL** |  | Welch ANOVA; Welch’s t-test |
|  | Mean ± SD, SEM | | | |  |  |
| **Small-sized Diameters** **(μm)** | 25.45 ± 3.47, 0.32 | 25.96 ± 3.78, 0.33 | 24.74 ± 3.16, 0.35 | 26.30 ± 4.14, 0.53 |  | p = 0.108 |
| **n** | 121 | 124 | 81 | 62 |  |  |
| **Large-sized Diameters** **(μm)** | 45.32 ± 5.28, 0.31 | 45.98 ± 5.30, 0.28 | 44.62 ± 5.20, 0.33 | 45.65 ± 4.84, 0.27 |  | MAL vs. GAL p = 0.009 |
| **n** | 298 | 352 | 252 | 310 |  |  |

# Table S13. SK3+ Motoneuron diameter sizes in galantamine treated groups

| **Male Immediate** | | | | | | |
| --- | --- | --- | --- | --- | --- | --- |
|  | **Saline** | **MAL** | **GAL** | **MAL + GAL** |  | **ANOVA; Dunn’s Method** |
|  | n = 137 | n = 123 | n = 108 | n = 88 |  |  |
|  | Mean ± SD, SEM | | | |  |  |
| **Diameters (μm)** | 42.29 ± 5.13, 0.44 | 43.15 ± 5.96, 0.54 | 41.30 ± 4.39, 0.42 | 40.97 ± 6.42, 0.68 |  | MAL vs. GAL p = 0.007 |
| **Male Delayed** | | | | | | |
|  | **Saline** | **MAL** | **GAL** | **MAL + GAL** |  | **ANOVA** |
|  | n = 118 | n = 155 | n = 84 | n = 98 |  |  |
|  | Mean ± SD, SEM | | | |  |  |
| **Diameters (μm)** | 43.50 ± 6.55, 0.60 | 44.19 ± 6.61, 0.53 | 42.92 ± 5.91, 0.65 | 43.98 ± 5.54, 0.56 |  | p = 0.444 |

# Table S14. SK3+ Motoneuron ratio in galantamine treated groups

| **Male Immediate** | | | | | | |
| --- | --- | --- | --- | --- | --- | --- |
|  | **Saline** | **MAL** | **GAL** | **MAL + GAL** |  | **ANOVA** |
|  | n = 4 | n = 4 | n = 3 | n = 3 |  |  |
|  | Mean ± SD, SEM | | | |  |  |
| **Ratio** | 0.31 ± 0.11, 0.05 | 0.19 ± 0.08, 0.04 | 0.26 ± 0.06, 0.03 | 0.22 ± 0.05, 0.03 |  | p = 0.298 |
| **Male Delayed** | | | | | | |
|  | **Saline** | **MAL** | **GAL** | **MAL + GAL** |  | **ANOVA** |
|  | n = 3 | n = 3 | n = 3 | n = 3 |  |  |
|  | Mean ± SD, SEM | | | |  |  |
| **Ratio** | 0.27 ± 0.10, 0.06 | 0.30 ± 0.07, 0.04 | 0.25 ± 0.05, 0.03 | 0.25 ± 0.04, 0.02 |  | p = 0.713 |

# Table S15. Average C-Bouton areas in galantamine treated groups

| **Male Immediate** | | | | | | |
| --- | --- | --- | --- | --- | --- | --- |
|  | **Saline** | **MAL** | **GAL** | **MAL + GAL** |  | **ANOVA** |
|  | n = 6 | n = 6 | n = 5 | n = 6 |  |  |
|  | Mean ± SD, SEM | | | |  |  |
| **Area (μm^2^)** | 11.27 ± 1.85, 0.76 | 10.45 ± 1.80, 0.74 | 11.70 ± 1.63, 0.73 | 11.98 ± 1.83, 0.75 |  | p = 0.496 |
| **Male Delayed** | | | | | | |
|  | **Saline** | **MAL** | **GAL** | **MAL + GAL** |  | **ANOVA** |
|  | n = 5 | n = 5 | n = 6 | n = 6 |  |  |
|  | Mean ± SD, SEM | | | |  |  |
| **Area (μm^2^)** | 11.08 ± 1.50, 0.67 | 10.67 ± 1.39, 0.62 | 12.82 ± 2.39, 0.98 | 11.23 ± 1.85, 0.75 |  | p = 0.257 |

# Table S16. Average C-bouton densities in galantamine treated groups

| **Male Immediate** | | | | | | |
| --- | --- | --- | --- | --- | --- | --- |
|  | **Saline** | **MAL** | **GAL** | **MAL + GAL** |  | **ANOVA** |
|  | n = 6 | n = 6 | n = 5 | n = 6 |  |  |
|  | Mean ± SD, SEM | | | |  |  |
| **Density (bouton/ μm^2^)** | 0.05 ± 0.01, 0.00 | 0.05 ± 0.01, 0.00 | 0.04 ± 0.01, 0.00 | 0.04 ± 0.01, 0.00 |  | p = 0.263 |
| **Male Delayed** | | | | | | |
|  | **Saline** | **MAL** | **GAL** | **MAL + GAL** |  | **ANOVA** |
|  | n = 5 | n = 5 | n = 6 | n = 6 |  |  |
|  | Mean ± SD, SEM | | | |  |  |
| **Density**  **(bouton/ μm^2^)** | 0.05 ± 0.00, 0.00 | 0.05 ± 0.01, 0.00 | 0.04 ± 0.01, 0.00 | 0.04 ± 0.01, 0.00 |  | p = 0.297 |

# Table S17. GFAP integrated optical density in galantamine treated groups

| **Male Immediate** | | | | | | |
| --- | --- | --- | --- | --- | --- | --- |
|  | **Saline** | **MAL** | **GAL** | **MAL + GAL** |  | **ANOVA** |
|  | n = 6 | n = 6 | n = 5 | n = 6 |  |  |
|  | Mean ± SD, SEM | | | |  |  |
| **Integrated Optical Density (lumμm^2^)** | 1.7x10^7^ ± 6.2x10^6^, 2.5x10^6^ | 1.8x10^7^ ± 7.8x10^6^, 3.2x10^6^ | 1.5x10^7^ ± 7.1x10^6^, 3.2x10^6^ | 1.7x10^7^ ± 1.1x10^7^, 4.6x10^6^ |  | p = 0.958 |
| **Male Delayed** | | | | | | |
|  | **Saline** | **MAL** | **GAL** | **MAL + GAL** |  | **ANOVA** |
|  | n = 5 | n = 5 | n = 6 | n = 6 |  |  |
|  | Mean ± SD, SEM | | | |  |  |
| **Integrated Optical Density (lumμm^2^)** | 1.7x10^7^ ± 3.7x10^6^, 1.7x10^6^ | 1.7x10^7^ ± 8.0x10^6^, 3.6x10^6^ | 1.6x10^7^ ± 6.1x10^6^, 2.5x10^6^ | 1.8x10^7^ ± 9.8x10^6^, 4.0x10^6^ |  | p = 0.977 |

# Table S18. GFAP mean intensity in galantamine treated groups

| **Male Immediate** | | | | | | |
| --- | --- | --- | --- | --- | --- | --- |
|  | **Saline** | **MAL** | **GAL** | **MAL + GAL** |  | **ANOVA** |
|  | n = 6 | n = 6 | n = 5 | n = 6 |  |  |
|  | Mean ± SD, SEM | | | |  |  |
| **Mean Intensity (lum)** | 33.07 ± 17.55, 7.16 | 32.55 ± 16.02, 6.54 | 23.90 ± 13.17, 5.89 | 29. 549 ± 21.20, 8.65 |  | p = 0.818 |
| **Male Delayed** | | | | | | |
|  | **Saline** | **MAL** | **GAL** | **MAL + GAL** |  | **ANOVA** |
|  | n = 5 | n = 5 | n = 6 | n = 6 |  |  |
|  | Mean ± SD, SEM | | | |  |  |
| **Mean Intensity (lum)** | 32.42 ± 8.54, 3.82 | 31.97 ± 13.08, 5.85 | 30.04 ± 13.61, 5.56 | 34.68 ± 14.52, 5.93 |  | p = 0.939 |

# Table S19. Immunohistochemistry antibodies and approach

| **Spinal Cord IHC** | | | | |
| --- | --- | --- | --- | --- |
| **Laser Excitation Channel** | **405** | **488** | **552** | **638** |
| **Primary Antibody** | **-** | **Mouse anti-KV2.1**  Antibodies Inc. 75-014  1:100 | **Rabbit anti-SK3**  EMD Millipore AB350  1:1000 | **Goat anti-VAchT**  EMD Millipore ABN100  1:1000 |
| **Secondary Antibody/Stain** | **Nissl blue**  Invitrogen N21479  1:100 | **Donkey anti-mouse 488**  Jackson IR 715-545-151  1:100 | **Donkey anti-rabbit CY3**  Jackson IR 711-165-152  1:100 | **Donkey anti-goat 647**  Jackson IR 705-605-147  1:100 |
|  | | | | |
| **Primary Antibody** | - | **Mouse anti-NeuN**  Millipore Sigma MAB377  1:500 | **Rabbit anti-SK3**  EMD Millipore AB5350  1:1000 | **Goat anti-VAchT**  EMD Millipore ABN100  1:1000 |
| **Secondary Antibody/Stain** | - | **Donkey anti-mouse 488**  Jackson IR 715-545-151  1:100 | **Donkey anti-Rabbit Cy3**  Jackson IR 711-165-152  1:100 | **Donkey anti-goat 647**  Jackson IR 705-605-147  1:100 |
|  | | | | |
| **Primary Antibody** | - | - | **Chicken anti-GFAP**  Invitrogen PA1-10004  1:1000 | - |
| **Secondary Antibody/Stain** | **DAPI**  Invitrogen cat# D3571  1:1000 | **Nissl Green**  Invitrogen N21480  1:100 | **Donkey anti-chicken Cy3**  Jackson IR 703-165-155  1:100 | - |
| **Whole Muscle IHC** | | | | |
| **Laser Excitation Channel** | **405** | **488** | **552** | **638** |
| **Primary Antibody** | - | - | **Rabbit anti-SV2B**  Cedarlane Labs 119102 (SY)  1:2000 | **Mouse anti-NF-H**  BioLegend 801601  1:500 |
| **Secondary Antibody/Stain** | - | **alpha-Bungarotoxin 488**  Molecular Probes B13422  1:1000 | **Donkey anti-rabbit Cy3**  Jackson IR 711-165-152  1:100 | **Donkey anti-mouse 647**  Jackson IR 715-605-151  1:100 |
