# Supplementary material for: Performance changes and neuromuscular alterations in sprague dawley rats following repeated occupational exposures to the pesticide malathion
Source: PLoS One. 2026 Sep 15;21(9):e0357900. doi: 10.1371/journal.pone.0357900 (PMC13577405; doi:10.1371/journal.pone.0357900)
Supplement: Peer review history

# Peer review: Decision Letter 0

**DOI:** 10.1371/journal.pone.0357900.r001

**Authors:** Keisuke Hitachi

30 Jul 2026

Dear Dr. Romer,

Thank you for submitting your manuscript to PLOS ONE. After careful consideration, we feel that it has merit but does not fully meet PLOS ONE’s publication criteria as it currently stands. Therefore, we invite you to submit a revised version of the manuscript that addresses the points raised during the review process.

==============================

Your manuscript was reviewed by one expert, who evaluated that this study presented highly plausible objectives and rationales. On the other hand, given the relatively small sample size and the substantial inter-individual variability observed within several groups, the authors should explicitly acknowledge these limitations in the Discussion.

==============================

Please submit your revised manuscript by Sep 13 2026 11:59PM. If you will need more time than this to complete your revisions, please reply to this message or contact the journal office at plosone@plos.org. When you're ready to submit your revision, log on to https://www.editorialmanager.com/pone/ and select the 'Submissions Needing Revision' folder to locate your manuscript file.

- A letter that responds to each point raised by the academic editor and reviewer(s). You should upload this letter as a separate file labeled 'Response to Reviewers'.
- A marked-up copy of your manuscript that highlights changes made to the original version. You should upload this as a separate file labeled 'Revised Manuscript with Track Changes'.
- An unmarked version of your revised paper without tracked changes. You should upload this as a separate file labeled 'Manuscript'.

If you would like to make changes to your financial disclosure, please include your updated statement in your cover letter. Guidelines for resubmitting your figure files are available below the reviewer comments at the end of this letter.

If applicable, we recommend that you deposit your laboratory protocols in protocols.io to enhance the reproducibility of your results. Protocols.io assigns your protocol its own identifier (DOI) so that it can be cited independently in the future. For instructions see: https://journals.plos.org/plosone/s/submission-guidelines#loc-laboratory-protocols. Additionally, PLOS ONE offers an option for publishing peer-reviewed Lab Protocol articles, which describe protocols hosted on protocols.io. Read more information on sharing protocols at https://plos.org/protocols?utm_medium=editorial-email&utm_source=authorletters&utm_campaign=protocols.

As the corresponding author, your ORCID iD is verified in the submission system and will appear in the published article. PLOS supports the use of ORCID, and we encourage all coauthors to register for an ORCID iD and use it as well. Please encourage your coauthors to verify their ORCID iD within the submission system before final acceptance, as unverified ORCID iDs will not appear in the published article. *Only*  the individual author can complete the verification step; PLOS staff *cannot*  verify ORCID iDs on behalf of authors.

We look forward to receiving your revised manuscript.

Kind regards,

Keisuke Hitachi

Academic Editor

PLOS One

Journal Requirements:

When submitting your revision, we need you to address these additional requirements.

1. Please ensure that your manuscript meets PLOS ONE's style requirements, including those for file naming. The PLOS ONE style templates can be found at

https://journals.plos.org/plosone/s/file?id=wjVg/PLOSOne_formatting_sample_main_body.pdf and

https://journals.plos.org/plosone/s/file?id=ba62/PLOSOne_formatting_sample_title_authors_affiliations.pdf

2. Thank you for stating the following in the Acknowledgments Section of your manuscript:

"The authors would like to thank the sponsor “The Office of Naval Research” for funding this project through the In-House Laboratory Independent Research program. The authors wish to express appreciation to the following individuals whose contributions helped made this project successful. Dr. Karen Mumy, Director of Environmental Health Effects Laboratory at the Naval Medical Research Unit Dayton for administrative and directorate support on this project. Heather Procopio, Josh Stricker, Elizabeth Philips, Hana Warwick, Nathan Gargas, Erin Roberts, and Victoria Ethridge for technical support on this project. Shawn McInturf, for neurobehavior testing support. Dr. Maria Spencer for discussions regarding the military relevance of this work. Wright Patterson Air Force Base Research Support Command for animal vivarium facilities support."

We note that you have provided additional information within the Acknowledgements Section that is not currently declared in your Funding Statement. Please note that funding information should not appear in the Acknowledgments section or other areas of your manuscript. We will only publish funding information present in the Funding Statement section of the online submission form.

Please remove any funding-related text from the manuscript and let us know how you would like to update your Funding Statement. Currently, your Funding Statement reads as follows:

"This work was funded by The Office of Naval Research through the In-House Laboratory Independent Research program."

Please include your amended statements within your cover letter; we will change the online submission form on your behalf.

3. Please note that your Data Availability Statement is currently missing a direct link to access each database. If your manuscript is accepted for publication, you will be asked to provide these details on a very short timeline. We therefore suggest that you provide this information now, though we will not hold up the peer review process if you are unable.

4. Please include captions for your Supporting Information files at the end of your manuscript, and update any in-text citations to match accordingly. Please see our Supporting Information guidelines for more information: http://journals.plos.org/plosone/s/supporting-information.

5. If the reviewer comments include a recommendation to cite specific previously published works, please review and evaluate these publications to determine whether they are relevant and should be cited. There is no requirement to cite these works unless the editor has indicated otherwise.

6. Please review your reference list to ensure that it is complete and correct. If you have cited papers that have been retracted, please include the rationale for doing so in the manuscript text, or remove these references and replace them with relevant current references. Any changes to the reference list should be mentioned in the rebuttal letter that accompanies your revised manuscript. If you need to cite a retracted article, indicate the article’s retracted status in the References list and also include a citation and full reference for the retraction notice.

[Note: HTML markup is below. Please do not edit.]

Reviewers' comments:

Reviewer's Responses to Questions

**Comments to the Author**

1. Is the manuscript technically sound, and do the data support the conclusions?

Reviewer #1: Partly

**********

2. Has the statistical analysis been performed appropriately and rigorously?

Reviewer #1: Yes

**********

3. Have the authors made all data underlying the findings in their manuscript fully available?

The PLOS Data policy

Reviewer #1: Yes

**********

4. Is the manuscript presented in an intelligible fashion and written in standard English?

Reviewer #1: Yes

**********

Reviewer #1: The study presents highly plausible objectives and rationales. The experiments conducted align with the proposed objectives, and the results are comprehensively presented and provide important information. However, the sample size is small, and the values observed in the animals within each group—including the saline group—show high heterogeneity. This is evident in the high standard deviation values and explains the presence of various "trends" across groups in the results. A larger sample size could better represent the impacts of chronic Malathion exposure on the studied population.

**********

what does this mean?). If published, this will include your full peer review and any attached files.

If you choose “no”, your identity will remain anonymous but your review may still be made public.

**Do you want your identity to be public for this peer review?**  For information about this choice, including consent withdrawal, please see our Privacy Policy

Reviewer #1: No

**********

[NOTE: If reviewer comments were submitted as an attachment file, they will be attached to this email and accessible via the submission site. Please log into your account, locate the manuscript record, and check for the action link "View Attachments". If this link does not appear, there are no attachment files.]

To ensure your figures meet our technical requirements, please review our figure guidelines: https://journals.plos.org/plosone/s/figures

You may also use PLOS’s free figure tool, NAAS, to help you prepare publication quality figures: https://journals.plos.org/plosone/s/figures#loc-tools-for-figure-preparation.

NAAS will assess whether your figures meet our technical requirements by comparing each figure against our figure specifications.

# Author comment: Author response to Decision Letter 1

**DOI:** 10.1371/journal.pone.0357900.r002

4 Aug 2026

Dear Reviewer,

On behalf of my co-authors, I would like to thank for your evaluation of our manuscript and for the opportunity to revise our work. We appreciate the positive assessment of the study's objectives, rationale, technical rigor, and overall presentation. The comments were constructive and have helped us improve the clarity of the manuscript.

In response to the reviewer's primary concern regarding the relatively modest animal sample size and the biological variability observed among animals, we have revised the manuscript to more explicitly acknowledge this limitation in the Discussion. We also expanded the Methods and Results sections to clarify our statistical approach. Specifically, we now emphasize that although approximately 4,300 motoneurons were analyzed to characterize the heterogeneous distribution of motoneuron phenotypes within each animal, statistical inference was performed using hierarchical mixed-effects and generalized estimating equation (GEE) models in which the individual animal, not the individual motoneuron, served as the independent experimental unit. These revisions clarify how the hierarchical structure of the data was incorporated into the statistical analyses while avoiding pseudoreplication.

We believe that these revisions have strengthened the manuscript and have fully addressed the concerns raised during peer review. We appreciate your time and consideration and respectfully submit the revised manuscript for further evaluation.

Sincerely,

Shannon H Romer, Ph.D.

Corresponding Author

Naval Medical Research Unit Dayton

Journal Requirements:

When submitting your revision, we need you to address these additional requirements.

1. Please ensure that your manuscript meets PLOS ONE's style requirements, including those for file naming. The PLOS ONE style templates can be found at

2. Thank you for stating the following in the Acknowledgments Section of your manuscript:

"The authors would like to thank the sponsor “The Office of Naval Research” for funding this project through the In-House Laboratory Independent Research program. The authors wish to express appreciation to the following individuals whose contributions helped made this project successful. Dr. Karen Mumy, Director of Environmental Health Effects Laboratory at the Naval Medical Research Unit Dayton for administrative and directorate support on this project. Heather Procopio, Josh Stricker, Elizabeth Philips, Hana Warwick, Nathan Gargas, Erin Roberts, and Victoria Ethridge for technical support on this project. Shawn McInturf, for neurobehavior testing support. Dr. Maria Spencer for discussions regarding the military relevance of this work. Wright Patterson Air Force Base Research Support Command for animal vivarium facilities support."

We note that you have provided additional information within the Acknowledgements Section that is not currently declared in your Funding Statement. Please note that funding information should not appear in the Acknowledgments section or other areas of your manuscript. We will only publish funding information present in the Funding Statement section of the online submission form.

Please remove any funding-related text from the manuscript and let us know how you would like to update your Funding Statement. Currently, your Funding Statement reads as follows:

"This work was funded by The Office of Naval Research through the In-House Laboratory Independent Research program."

Please include your amended statements within your cover letter; we will change the online submission form on your behalf.

Response: The funding statement has been removed from Acknowledgements and the current funding statement is sufficient.

3. Please note that your Data Availability Statement is currently missing a direct link to access each database. If your manuscript is accepted for publication, you will be asked to provide these details on a very short timeline. We therefore suggest that you provide this information now, though we will not hold up the peer review process if you are unable.

Response: The link provided is now active and will take the readers to publicly available datasets.

4. Please include captions for your Supporting Information files at the end of your manuscript, and update any in-text citations to match accordingly. Please see our Supporting Information guidelines for more information: http://journals.plos.org/plosone/s/supporting-information.

Response: Captions for Supporting Information Files have been moved to the end of the manuscript.

5. If the reviewer comments include a recommendation to cite specific previously published works, please review and evaluate these publications to determine whether they are relevant and should be cited. There is no requirement to cite these works unless the editor has indicated otherwise.

Response: No changes to citations were made.

6. Please review your reference list to ensure that it is complete and correct. If you have cited papers that have been retracted, please include the rationale for doing so in the manuscript text, or remove these references and replace them with relevant current references. Any changes to the reference list should be mentioned in the rebuttal letter that accompanies your revised manuscript. If you need to cite a retracted article, indicate the article’s retracted status in the References list and also include a citation and full reference for the retraction notice.

Response: To our knowledge, the references appear accurate

Reviewers' comments:

Please use the space provided to explain your answers to the questions above. You may also include additional comments for the author, including concerns about dual publication, research ethics, or publication ethics. (Please upload your review as an attachment if it exceeds 20,000 characters)

Reviewer #1: The study presents highly plausible objectives and rationales. The experiments conducted align with the proposed objectives, and the results are comprehensively presented and provide important information. However, the sample size is small, and the values observed in the animals within each group—including the saline group—show high heterogeneity. This is evident in the high standard deviation values and explains the presence of various "trends" across groups in the results. A larger sample size could better represent the impacts of chronic Malathion exposure on the studied population.

Response

We sincerely thank the reviewer for the thoughtful comments and positive assessment of our study. We agree that biological variability was present among animals, including within the saline control groups, and we acknowledge that larger cohorts can improve the precision of estimated treatment effects. We have revised the manuscript to more clearly discuss this limitation in the Discussion and to better explain our statistical approach in the Methods.

Importantly, the motoneuron analyses were designed to characterize a biologically heterogeneous neuronal population rather than to estimate a single mean value per animal. Motoneurons comprise multiple functional subtypes whose soma sizes, recruitment thresholds, electrophysiological properties, and motor unit characteristics exist along a continuum rather than as a single homogeneous population. Moreover, selective vulnerability of specific motoneuron subpopulations is a well-recognized feature of several motor neuron disorders, where larger motoneurons often exhibit greater susceptibility to degeneration than smaller motoneurons. Consequently, reducing each animal to a single average motoneuron diameter would obscure biologically meaningful differences in the distribution of motoneuron phenotypes and could mask selective effects on particular subpopulations.

For this reason, approximately 4,300 motoneurons were sampled across 32 animals to provide a representative characterization of the motoneuron population within each animal. However, statistical inference was not based on the individual neuronal measurements. Because multiple neurons were measured from each animal, the data possess a hierarchical structure, and we analyzed these data using linear mixed-effects models with animal included as a random effect and complementary generalized estimating equation (GEE) models. These methods explicitly account for the correlation among neurons originating from the same animal while estimating treatment effects at the animal level, thereby avoiding pseudoreplication and appropriately incorporating inter-animal variability into the statistical inference.

We agree that the relatively modest number of animals per group may have reduced statistical power to detect smaller treatment effects, particularly in the presence of normal biological variability. Accordingly, we have revised the Discussion to acknowledge this limitation and note that replication in larger cohorts would further refine estimates of effect size and improve precision. Nevertheless, statistically significant treatment-related differences were detected despite the observed biological heterogeneity, supporting the principal conclusions of the study while recognizing the value of future studies with larger sample sizes.

To further explain the logic for the use of the selected statistical test and to provide more clarification, we added the following to the Methods section:

“Because MN soma size spans a continuum of phenotypes rather than representing a homogeneous population, multiple MNs were sampled from each animal to characterize the distribution of MN sizes within each animal. Consequently, the individual animal, rather than the individual MN, served as the independent experimental unit for statistical inference, while within-animal correlation among MNs was accounted for analytically.”

To remind the readers that statistical inference comes from animal level effects, we added the following sentence to the Results section:

“Because multiple neurons were sampled from each animal, statistical inference was performed using mixed-effects models that accounted for clustering within animals rather than treating individual neurons as independent observations.”

To Discussion we added the following paragraph:

“A limitation of this study is the relatively modest number of animals within each sex and treatment group, which may have reduced sensitivity for detecting subtle treatment effects in the presence of normal biological variability. However, MNs represent a biologically heterogeneous population whose soma sizes span a continuum, therefore, large numbers of neurons were sampled from each animal to characterize the distribution of MN phenotypes rather than to increase the effective sample size. Statistical inference was performed using hierarchical mixed-effects and GEE models in which the individual animal, rather than individual MNs, served as the experimental unit, thereby accounting for within-animal correlation while avoiding pseudoreplication. Future studies including larger animal cohorts will be valuable to further refine effect size estimates and assess the reproducibility of these findings.”

# Peer review: Decision Letter 1

**DOI:** 10.1371/journal.pone.0357900.r003

**Authors:** Keisuke Hitachi

24 Aug 2026

Performance Changes and Neuromuscular Alterations in Sprague Dawley Rats Following Repeated Occupational Exposures to the Pesticide Malathion

PONE-D-26-18436R1

Dear Dr. Romer,

We’re pleased to inform you that your manuscript has been judged scientifically suitable for publication and will be formally accepted for publication once it meets all outstanding technical requirements.

Within one week, you’ll receive an e-mail detailing the required amendments. When these have been addressed, you’ll receive a formal acceptance letter and your manuscript will be scheduled for publication.

An invoice will be generated when your article is formally accepted. Please note, if your institution has a publishing partnership with PLOS and your article meets the relevant criteria, all or part of your publication costs will be covered. Please make sure your user information is up-to-date by logging into Editorial Manager at Editorial Manager® and clicking the ‘Update My Information' link at the top of the page. For questions related to billing, please contact billing support.

If your institution or institutions have a press office, please notify them about your upcoming paper to help maximize its impact. If they’ll be preparing press materials, please inform our press team as soon as possible -- no later than 48 hours after receiving the formal acceptance. Your manuscript will remain under strict press embargo until 2 pm Eastern Time on the date of publication. For more information, please contact onepress@plos.org.

Kind regards,

Keisuke Hitachi

Academic Editor

PLOS One

Additional Editor Comments (optional):

Reviewers' comments:

Reviewer's Responses to Questions

**Comments to the Author**

Reviewer #1: All comments have been addressed

**********

2. Is the manuscript technically sound, and do the data support the conclusions?

Reviewer #1: Yes

**********

3. Has the statistical analysis been performed appropriately and rigorously?

Reviewer #1: Yes

**********

4. Have the authors made all data underlying the findings in their manuscript fully available?

The PLOS Data policy

Reviewer #1: Yes

**********

5. Is the manuscript presented in an intelligible fashion and written in standard English?

Reviewer #1: Yes

**********

Reviewer #1: The authors accepted the suggestion and presented this limitation more clearly in the Discussion section; they also described the statistical approach used in greater detail in the Methods section.

**********

what does this mean?). If published, this will include your full peer review and any attached files.

If you choose “no”, your identity will remain anonymous but your review may still be made public.

**Do you want your identity to be public for this peer review?**  For information about this choice, including consent withdrawal, please see our Privacy Policy

Reviewer #1: No

**********

# Editor report: Acceptance letter

**DOI:** 10.1371/journal.pone.0357900.r004

**Authors:** Keisuke Hitachi

PONE-D-26-18436R1

PLOS One

Dear Dr. Romer,

I'm pleased to inform you that your manuscript has been deemed suitable for publication in PLOS One. Congratulations! Your manuscript is now being handed over to our production team.

At this stage, our production department will prepare your paper for publication. This includes ensuring the following:

* All references, tables, and figures are properly cited

* All relevant supporting information is included in the manuscript submission,

* There are no issues that prevent the paper from being properly typeset

You will receive further instructions from the production team, including instructions on how to review your proof when it is ready. Please keep in mind that we are working through a large volume of accepted articles, so please give us a few days to review your paper and let you know the next and final steps.

Lastly, if your institution or institutions have a press office, please let them know about your upcoming paper now to help maximize its impact. If they'll be preparing press materials, please inform our press team within the next 48 hours. Your manuscript will remain under strict press embargo until 2 pm Eastern Time on the date of publication. For more information, please contact onepress@plos.org.

You will receive an invoice from PLOS for your publication fee after your manuscript has reached the completed accept phase. If you receive an email requesting payment before acceptance or for any other service, this may be a phishing scheme. Learn how to identify phishing emails and protect your accounts at https://explore.plos.org/phishing.

If we can help with anything else, please email us at customercare@plos.org.

Thank you for submitting your work to PLOS One and supporting open access.

Kind regards,

PLOS One Editorial Office Staff

on behalf of

Dr. Keisuke Hitachi

Academic Editor

PLOS One
